# “Smelltronics”—From Gas to Smell Sensing

**DOI:** 10.1002/adma.202523677

**Published:** 2026-08-28

**Authors:** Takeshi Ono, Tsunaki Takahashi, Jiangyang Liu, Haruka Honda, Zeng Jing, Wataru Tanaka, Takuro Hosomi, Takeshi Yanagida

**Affiliations:** ^1^ Department of Applied Chemistry Graduate School of Engineering The University of Tokyo Bunkyo Tokyo Japan; ^2^ Institute For Materials Chemistry and Engineering Kyushu University Kasuga Fukuoka Japan

**Keywords:** gas sensors, interfaces, sensing materials, sensor array, smell sensing, smelltronics

## Abstract

This review discusses recent trends in smell and odor‐sensing studies, including materials, devices, and interfaces. Since the invention of chemical sensors, particularly over the past few decades, gas sensor materials and devices for small molecules have been extensively investigated. Recently, the social value of odor information has been strongly reaffirmed across diverse research fields, triggered by the rapid development of artificial intelligence (AI) and machine learning (ML). Compared with conventional gas sensing, the requirements for sensing materials and devices for extracting odor information are fundamentally different. In the fields of health diagnosis, food inspection, and environmental monitoring, target molecules that express odor information are frequently composed of molecules with relatively large molecular weights. Because the structural isomers of such relatively large molecules essentially exhibit variations in odor information, their discrimination of such structural isomers is an important issue for smell/odor sensing. Various sensors that operate based on different principles, including electrical, gravimetric, and optical sensors, were discussed in terms of their characteristics. In addition, we describe the sensing materials and interfaces for smell/odor sensing–*smelltronics*. Finally, we discuss the current challenges and perspectives of smelltronics.

## Graphical abstract

The emerging field of smelltronics, encompassing sensing technologies for complex volatile organic compounds, holds significant potential for extracting valuable chemical information. It facilitates the noninvasive, real‐time monitoring of humans, food, and the environment. This review examines the sensing materials, devices, and systems employed for odor detection, addressing current challenges and future perspectives.

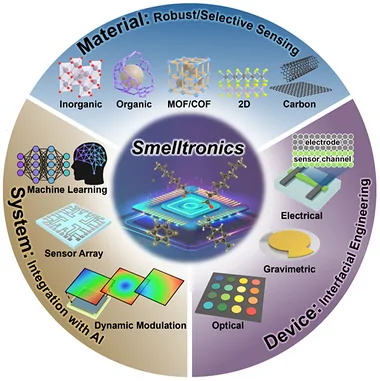

## 1 Introduction

Information science, which has been rapidly developing recently, is enhancing the value and meaning of sensing various types of information surrounding us [1, 2, 3, 4]. Among various chemical information data, odor information has attracted much attention due to its non‐invasiveness and diversity of information content, and the value of odor information has been reaffirmed in various research fields [5, 6, 7, 8]. Compared with various physical information data, including images and sounds, which are often in the form of wave information, chemical information data are mainly based on the diversity of molecular structures with multiple isomers. Such structural variation in molecules inherently creates the value and diversity of odor information, as can be seen in biological systems [9, 10, 11, 12, 13, 14, 15]. Although gas sensing for relatively small molecules, including H_2_, H_2_O, CH_4_, CO, CO_2_, NH_3_, NO, NO_2_, O_2_, H_2_S, SO_2_, and others, has been well developed and investigated to measure specific molecules in the surroundings [16, 17, 18, 19, 20, 21, 22], the requirements of sensing materials and devices to extract odor information inherently differ from those of conventional gas sensing technology because of the different target molecules, which are composed of molecules with relatively large molecular weights. In the natural world, biological olfaction effectively extracts multidimensional information. Human and animal olfactory receptors utilize weak noncovalent interactions, such as van der Waals forces, to selectively identify the diverse carbon skeletons of odorant volatile organic compounds (VOCs), which typically include molecules with molecular weights up to approximately 300 Da [23, 24, 25, 26]. However, the outputs of biological olfaction are heavily dependent on physiological reactions or subjective language, rendering them inherently challenging to handle objectively, express quantitatively, or digitize for seamless integration into contemporary information networks [27, 28]. Thus, there is still debate regarding the type of sensing materials, devices, and sensing interfaces required to fully extract the value of odor information objectively and quantitatively. In this article, we examine recent trends in smell and odor sensing research, collectively termed “*smelltronics*,” a distinct paradigm that aims to sense larger VOCs and bridge the gap between conventional gas sensing and biological olfaction (Table 1). Smelltronics endeavors to actively employ weak interactions to achieve the sophisticated molecular recognition capabilities of biological systems while simultaneously providing the robustness, reproducibility, and digitizability inherent to artificial devices. Traditional electronic noses (e‐noses) predominantly rely on system‐level pattern recognition algorithms in conjunction with cross‐reactive sensor arrays. In contrast, smelltronics employs a materials science‐oriented methodology that emphasizes the rational design of sensing interfaces to directly capture odor information or enhance molecular recognition before algorithmic processing. Various sensors that operate on different principles, including electrical sensors (chemiresistors, chemicapacitors, electrochemical sensors, and field‐effect transistors (FETs)), gravimetric sensors (quartz crystal microbalance (QCM) and surface acoustic wave (SAW)), and optical sensors (colorimetric, fluorescent, and plasmonic sensors), have been discussed and compared in terms of their features and characteristics for smell detection. In addition, various sensing materials and interfaces, including metals, metal oxides, organic molecules, polymers, covalent organic frameworks (COFs), metal–organic frameworks (MOFs), 2D materials and others, have been compared for smell‐sensing applications. Finally, we discuss the current issues and perspectives of smelltronics for volatile chemical sensing (Figure 1).

**TABLE 1:** Comparison table of conventional gas sensing, smelltronics, and biological olfaction. b.p., boiling point; v.p., vapor pressure.

	Biological olfaction	Conventional gas sensing	Smelltronics
System category	Biological	Artificial	Artificial
Target molecules	Odor molecules (VOCs < 300 Da)	Small gases (e.g., H_2_, CO, NH_3_, and H_2_S)	Informative VOCs (e.g., aldehydes, aromatics, and terpenes)
Physicochemical boundary	N/A (volatile chemical species that have odor)	b.p. ≤ 25°C, v.p. ≥ 100 kPa at 25°C (or non‐condensable gas)	b.p. > 25°C, v.p. < 100 kPa at 25°C
Sensing methodology	Utilizing weak interactions between odor molecules and olfactory receptors	Strong chemisorption, acid‐base, redox reactions	Amplification of weak noncovalent interactions (e.g., van der Waals interaction)
Output	Perception of odor information through combinatorial coding (flavor, danger identification)	Digitized and quantifiable information, concentration of a specific target	Digitized and quantifiable information, multidimensional “odor space”
Key advantages	Ultra‐high sensitivity and selectivity, ultra‐low power, dynamic learning	High robustness, simple readout, operable in harsh conditions	Digitizable/transmittable data, continuous monitoring, scalability, tailored selectivity
Intrinsic limitations	Non‐digitizable, olfactory fatigue, working environments, training cost, ethical constraints	Poor sensitivity low‐reactive VOCs, poor selectivity toward structurally similar VOCs	Inherent trade‐off between distinctive recognition and material robustness

**FIGURE 1:**
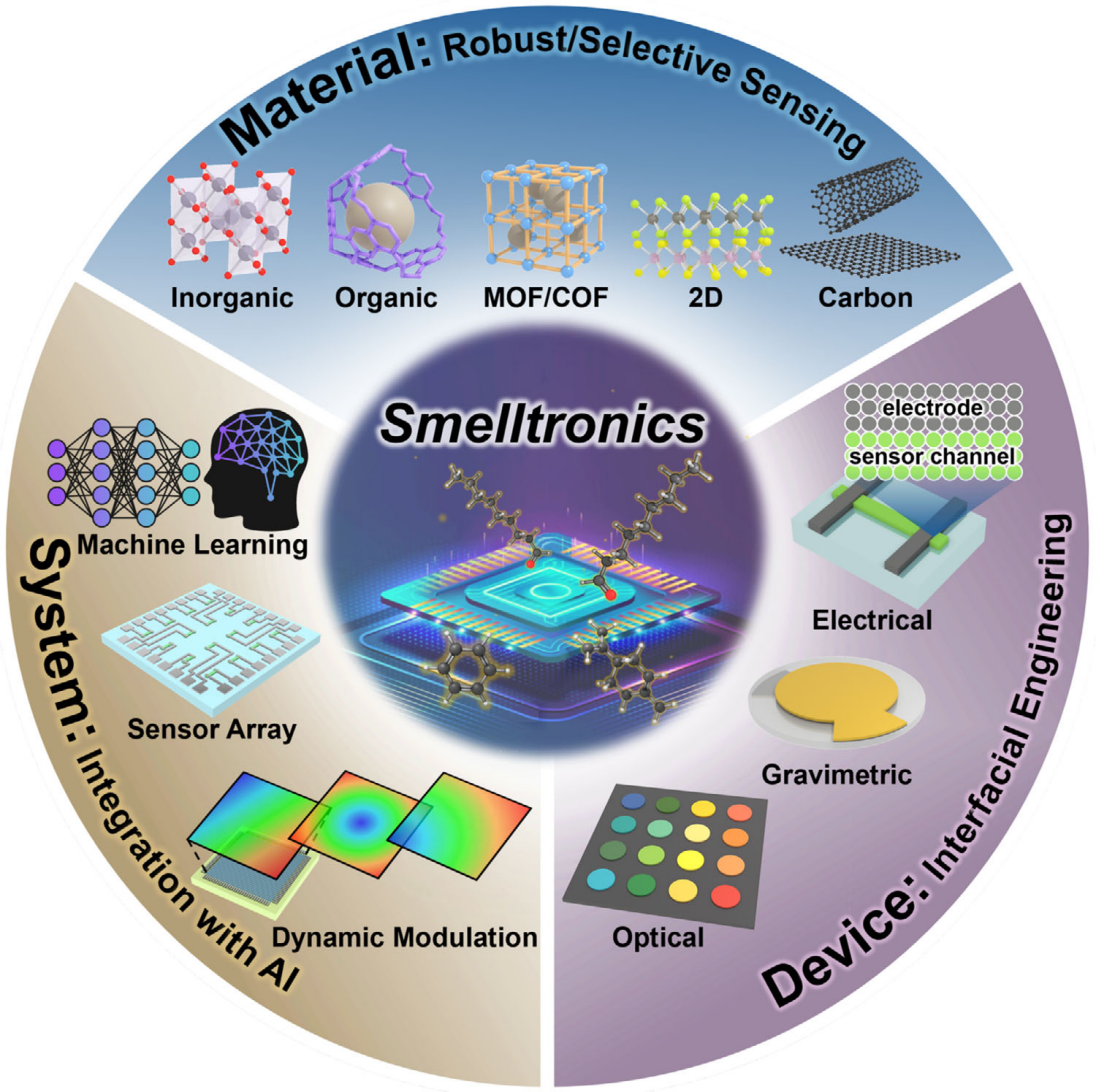
Conceptual overview of smelltronics. The domain of chemical sensing is undergoing a transformation from traditional gas sensing, which predominantly focuses on highly volatile gases, to smelltronics, which seeks to detect and differentiate complex VOCs that convey specific odor information. To achieve precise odor identification, smelltronics relies on the development of three hierarchies: (1) diverse sensing materials engineered for specific interactions, (2) devices that transduce physicochemical events into digitized signals, and (3) systems that integrate sensor arrays with information science.

## 2 Importance of Large Volatile Molecules on Odor Information

Numerous studies have proposed various chemical sensors capable of detecting gas molecules such as H_2_, CO, NO, NO_2_, NH_3_, H_2_S, and SO_2_, to assess air quality and identify potential hazards [16, 29]. Although detection techniques for these small gas species are important, recent research has highlighted the importance of sensing VOCs containing multiple carbon atoms. Given that living organisms, including microorganisms [30], plants [31], insects [32], and humans [5, 33], emit substantial amounts of VOCs because of their metabolic processes, these volatile molecules hold considerable potential for monitoring and influencing the physiological states of these organisms. Consequently, the sensitive, selective, rapid, and reliable detection of VOCs with a relatively large number of carbon atoms is promising for enabling real‐time and noninvasive evaluations, such as health diagnosis, food inspection, and environmental monitoring (Table 2). Such VOCs are also instrumental in enhancing public safety and security through the early detection of explosives and chemical warfare agents [34]. Furthermore, the dependency of VOC patterns emitted by individuals indicates their potential utility in personal authentication. This chemical information is inherently complex, dynamic, and challenging to replicate, making it a promising candidate for secure personal identification [35]. However, several challenges must be addressed to facilitate the detection of VOCs in real‐world applications. For example, the concentrations of these volatile molecules in the gas phase are low [34, 36, 37, 38]. The minimal quantities of these gaseous analytes and their low volatility, which is attributed to their larger molecular weights, hinder their sensitive detection. Based on their physicochemical properties, the sensing targets of smelltronics differ significantly from those of conventional gas sensing. Conventional gas sensors are typically designed to detect small, highly volatile gases characterized by boiling points of ≤ 25°C and vapor pressures of ≥ 100 kPa at 25°C. Conversely, smelltronics is specifically designed to target VOCs with boiling points exceeding 25°C and vapor pressures below 100 kPa at 25°C. In other words, it focuses on chemical species that exist in a vapor–liquid or solid–vapor equilibrium state under ambient pressure and temperature. Table 3 highlights a representative group of these VOCs, which are anticipated to be marker molecules reflecting human and plant health, food ripening and spoilage, and environmental pollution. Additionally, the chemical similarity of VOCs limits selective sensing; distinguishing the carbon skeletons of molecules with identical functional groups is challenging, yet essential for the precise identification and quantification of specific VOCs in realistic mixtures [39]. Interactions of varying strengths between sensing materials and analytes can be employed to detect VOCs [40]. Nonetheless, recognizing differences in carbon skeletons relies on weak interactions, such as van der Waals forces. In contrast, functional groups can strongly interact with the sensing materials, obscuring the weak interactions and subtle differences in the chemical structures of VOCs (Figure 2a). Similar to the manner in which biological olfactory receptors differentiate structurally similar odorants through tight packing interactions, smelltronics must meticulously design sensing interfaces to exploit these subtle interactions. Discrimination of isomers, such as diastereomers or enantiomers, is required in the most challenging cases. Therefore, VOC sensors capable of sensitively detecting analytes at parts per billion (ppb) levels and discriminating between similar structures are necessary for sensing VOCs in realistic environments and extracting valuable information. Here, we introduce representative VOCs that serve as meaningful indicators and have promising applications in health diagnosis, food inspection, and environmental monitoring.

**TABLE 2:** Summary of the classes of informative VOCs and their potential applications, including health diagnosis, food inspection, and environmental monitoring.

	C1–C3	C4–C6	C7–C9	C10–
Alcohol/phenol	Food inspection (meat spoilage [10], apricot [41])	Health diagnosis (gastric cancer [42], breast cancer [42], human body odor [43, 44]), food inspection (meat spoilage [10], banana [41], apple [41], grape [41], strawberry [41], peach [45], apricot [41], pineapple [41]), environment monitoring (biomass burning [6], microbial [46], plant [47, 48], soil [49, 50])	Health diagnosis (asthma [42], chronic obstructive pulmonary disease [42], human body odor [43, 44]), food inspection (meat spoilage [10], grape [41], strawberry [41], citrus [41]), environment monitoring (cable fire detection [51, 52]), environment monitoring (microbial [46], plant [48, 53], soil [49, 50])	Health diagnosis (human body odor [43, 44]), food inspection (meat spoilage [10], grape [41], strawberry [41], citrus [41], mango [41], peach [41], apricot [41]), environment monitoring (plant [47, 48], insect [32], soil [49, 50])
Aldehyde	Environment monitoring (petrochemical process [6], diesel vehicular emission [6], natural gas combustion [6], biomass burning [6])	Health diagnosis (lung cancer [9, 42]), food inspection (meat spoilage [10], banana [41], apple [41], grape [41], strawberry [41], mango [41], peach [45], apricot [41]), environment monitoring (biomass burning [6], plant [48, 54])	Health diagnosis (lung cancer [9]), food inspection (meat spoilage [10], grape [41], strawberry [41], citrus [41], mango [41], peach [41, 45], apricot [41]), environment monitoring (biomass burning [6], microbial [46], plant [48], soil [49])	Health diagnosis (lung cancer [42]), food inspection (meat spoilage [10], grape [41], citrus [41], mango [41], apricot [41], pineapple [41]), environment monitoring (plant [48], insect [32], soil [49, 50])
Ketone	Environment monitoring (concentrated animal feeding operations including animal exhalation [6])	Health diagnosis (lung cancer [42]), food inspection (meat spoilage [10], banana [41], strawberry [41], pineapple [41]), environment monitoring (microbial [46], plant [47], soil [49])	Food inspection (meat spoilage [10], strawberry [41], mango [41], apricot [41], pineapple [41]), environment monitoring (microbial [46], plant [48], insect [32], soil [49, 50], cable fire detection [52])	Health diagnosis (human body odor [43, 44]), food inspection (meat spoilage [10], citrus [41], mango [41], apricot [41]), environment monitoring (microbial [46], spoil [50])
Carboxylic acid	Health diagnosis (lung cancer [42]), food inspection (meat spoilage [10], pineapple [41]), environment monitoring (microbial [46], soil [49])	Health diagnosis (breast cancer [42]), food inspection (meat spoilage [10], grape [41]), environment monitoring (microbial [46], soil [49])	Health diagnosis (colorectal cancer [42], asthma [42], obstructive sleep apnea [42], human body odor [43, 44]), food inspection (meat spoilage [10], banana [41], grape [41]), environment monitoring (plant [47], soil [50])	Health diagnosis (colorectal cancer [42], asthma [42], human body odor [43, 44]), food inspection (meat spoilage [10], strawberry [41], apricot [41]), environment monitoring (microbial [46])
Ester		Food inspection (meat spoilage [10], apple [41], grape [41], strawberry [41], citrus [41], mango [41], peach [45], apricot [41], pineapple [41]), environment monitoring (microbial [46], soil [50])	Food inspection (meat spoilage [10], banana [41], apple [41], grape [41], strawberry [41], mango [41], peach [45], apricot [41], pineapple [41]), environment monitoring (microbial [46], plant [47, 48], soil [49])	Food inspection (meat spoilage [10], apple [41], grape [41], strawberry [41], apricot [41], pineapple [41]), environment monitoring (cable fire detection [51], insect [32], soil [50])
Amine	Food inspection (food spoilage [55, 56])	Food inspection (food spoilage [55, 56])	Food inspection (food spoilage [55, 56]), environment monitoring (soil [50])	Food inspection (food spoilage [55])
Aliphatic hydrocarbon	Environment monitoring (petroleum refinery [6], natural gas combustion [6], concentrated animal feeding operations including animal exhalation [6])	Environment monitoring (petroleum distillates [6], gasoline vehicular emission [6], petroleum refinery [6], natural gas combustion [6], concentrated animal feeding operations including animal exhalation [6])	Food inspection (grape [41]), environment monitoring (petroleum distillates [6], gasoline vehicular emission [6])	Food inspection (grape [41]), environment monitoring (petroleum distillates [6], insect [32])
Aromatic hydrocarbon		Environment monitoring (petrochemical process [6], diesel vehicular emission [6], natural gas combustion [6])	Environment monitoring (petrochemical process [6], petroleum distillates [6], gasoline vehicular emission [6], natural gas combustion [6])	Food inspection (grape [41]), environment monitoring (natural gas combustion [6])
Terpene/terpenoid				Food inspection (apple [41], grape [41], citrus [41], mango [41], peach [45], apricot [41]), environment monitoring (fragrances [6], plant [47, 48, 57], soil [49])

**TABLE 3:** Comparison table of conventional gas sensing and smelltronics targets. Physicochemical properties were obtained from the PubChem database [58].

	Molecule	Formula	Molecular weight (Da)	Boiling point(°C)	Vapor pressure at 25°C (kPa)
Conventional gas targets	Hydrogen	H_2_	2.02	−253	Non‐condensable
	Water (Humidity)	H_2_O	18.02	100	3.2
	Methane	CH_4_	16.04	−161	Non‐condensable
	Carbon monoxide	CO	28.01	−191	Non‐condensable
	Carbon dioxide	CO_2_	44.01	−78	6400
	Ammonia	NH_3_	17.03	−33	1000
	Nitric oxide	NO	30.01	−151	Non‐condensable
	Nitrogen dioxide	NO_2_	46.01	21	100
	Oxygen	O_2_	32.00	−183	Non‐condensable
	Hydrogen sulfide	H_2_S	34.08	−60	2100
	Sulfur dioxide	SO_2_	64.07	−10	400
Smelltronics targets	1‐Butanol	C_4_H_10_O	74.12	117	0.93
	1‐Hexanol	C_6_H_14_O	102.17	157	0.12
	Hexanal	C_6_H_12_O	100.16	130	1.5
	Nonanal	C_9_H_18_O	142.24	195	0.049
	Benzaldehyde	C_7_H_6_O	106.12	178	0.17
	Acetone	C_3_H_6_O	58.08	56	30
	Hexanoic acid	C_6_H_12_O_2_	116.16	205	0.0053
	Ethyl acetate	C_4_H_8_O_2_	88.11	77	12
	Isobutyl acetate	C_6_H_12_O_2_	116.16	117	2.4
	Triethylamine	C_6_H_15_N	101.19	89	7.6
	Cadaverine	C_5_H_14_N_2_	102.18	179	0.13
	Hexane	C_6_H_14_	86.18	69	20
	Benzene	C_6_H_6_	78.11	80	13
	Toluene	C_7_H_8_	92.14	110	3.8
	*p*‐Xylene	C_8_H_10_	106.16	138	1.2
	Isoprene	C_5_H_8_	68.12	34	73
	Limonene	C_10_H_16_	136.23	177	0.21
	α‐Pinene	C_10_H_16_	136.23	156	0.63
	Linalool	C_10_H_18_O	154.25	198	0.021

**FIGURE 2:**
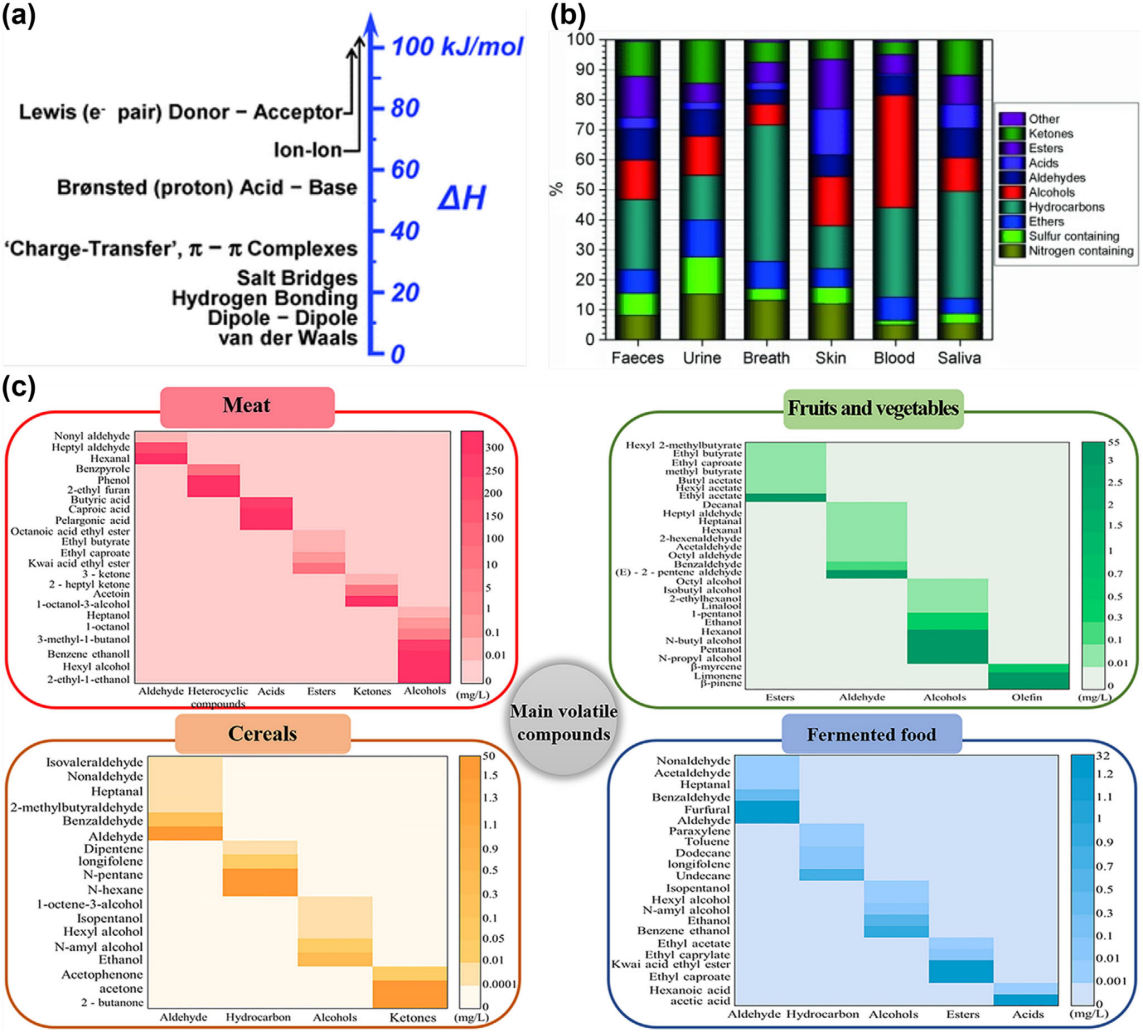
(a) The spectrum of interactions between the sensing materials and VOCs is depicted on a semi‐quantitative energy scale. These interactions, which are pertinent to VOC detection, range from weak van der Waals forces to strong covalent and ionic bonds. Reproduced with permission [40]. Copyright 2013, Royal Society of Chemistry. (b) The relative abundance of compounds within each chemical class detected in typical biofluids, which hold potential for real‐time and noninvasive disease diagnosis. Reproduced with permission [37]. Copyright 2015, Wiley. (c) VOCs emitted from various foods and their corresponding chemical classes. Reproduced with permission [120]. Copyright 2022, Taylor & Francis.

### 2.1 Health Diagnosis

Humans emit over 1800 VOCs from various biofluids, including breath, skin, feces, saliva, urine, and blood, which serve as rich, noninvasive biomarkers for a range of diseases and disorders [8, 37, 59, 60, 61]. Various VOCs, such as hydrocarbons, alcohols, aldehydes, ketones, acids, and esters, have been identified as emissions from the human body (Figure 2b) [37, 62, 63]. The continuous monitoring of these volatile signatures offers a transformative approach for early‐stage disease diagnosis and personalized medicine. However, extracting clinically actionable information from these biomarkers presents significant challenges owing to their trace concentrations (often in the ppb or parts per trillion (ppt) range), chemical similarity, and the overwhelming complexity of biological matrices. For instance, nonpolar hydrocarbons [64, 65, 66, 67] such as isoprene (a well‐known marker of muscle metabolism) [68, 69] and terpenes such as limonene (a recognized marker for liver cirrhosis) [70, 71] are notoriously difficult to detect selectively. They are typically exhaled at low ppb levels and lack highly reactive functional groups. This chemical inertness makes them susceptible to severe interference from highly concentrated polar molecules inherently present in breath, such as acetone, ammonia, or saturated water vapor [72, 73, 74, 75]. In contrast, oxygenated VOCs such as acetone have been more extensively monitored, primarily for diabetes diagnosis, with concentrations generally exceeding 1.7 ppm in patients compared to less than 0.8 ppm in healthy individuals [76, 77, 78, 79]. Recently, larger VOCs with multiple carbon atoms have gained increasing attention as potential indicators of disease. Straight‐chain aliphatic aldehydes (e.g., pentanal to nonanal), which originate from lipid peroxidation pathways under oxidative stress, are emerging as critical biomarkers for lung cancer and other oxidative‐stress‐related diseases [9, 13, 80, 81, 82, 83, 84, 85]. Other carbonyl compounds, including larger ketones and carboxylic acids, have also been intrinsically linked to specific cancers and metabolic disorders [81, 86, 87, 88, 89, 90]. Although gas chromatography–mass spectrometry (GC‐MS) has elucidated the existence and concentration variations of the aforementioned VOCs, the intricate nature of these physiological matrices poses significant challenges for conventional gas sensors that aim to selectively detect specific biomarkers. To overcome this limitation and address the structural similarities among diagnostically relevant VOCs, researchers have increasingly adopted cross‐reactive sensor arrays coupled with advanced pattern recognition techniques inspired by biological olfactory systems [3, 59, 60, 91, 92, 93]. Rather than targeting a single molecule with high specificity, which is often unfeasible in complex gas mixtures, these arrays generate multidimensional response patterns. For example, Nakhleh et al. successfully classified 17 different diseases using a highly sensitive chemiresistor array based on functionalized gold nanoparticles and carbon nanotubes (CNTs), highlighting how capturing “disease fingerprints” from exhaled breath combined with machine learning (ML) algorithms can overcome the limitations of individual sensors [59].

### 2.2 Food Inspection

VOC detection techniques are indispensable for food inspection due to their role in forming complex and diverse odor and flavor profiles [94, 95]. Additionally, as various plants, fruits, and insects emit distinct VOC signatures dynamically based on their physiological conditions, these volatiles enable continuous monitoring and non‐destructive management of agricultural products throughout the supply chain [96, 97, 98, 99, 100, 101]. Consequently, VOC detection techniques have been utilized to evaluate flavor [102, 103, 104, 105], ripening [106, 107, 108, 109, 110], fermentation [111, 112], insect infestation [96, 98, 113, 114], and spoilage [115, 116, 117, 118, 119] in a wide range of food products. The sensory qualities of foods and beverages are primarily determined by complex, multicomponent mixtures of esters, alcohols, aldehydes, and carboxylic acids (Figure 2c) [120]. Given that even subtle structural variations, such as carbon chain length or functional group positioning, can impart entirely different sensory impressions and flavor profiles [104, 105, 121], cross‐reactive sensor arrays are highly effective for comprehensive quality assessment. For instance, Li et al. employed a metal‐oxide sensor array to classify Chinese baijiu—a highly intricate beverage containing over 2000 distinct VOCs—based entirely on the multidimensional response profiles of its distinct VOC classes [105]. Beyond nuanced flavor profiling, specific VOCs serve as highly targeted indicators of biological degradation and bacterial contamination. Biogenic aliphatic amines, primarily produced via amino acid decarboxylation during microbial growth, are established and reliable biomarkers for food spoilage [122, 123, 124, 125]. The sum of these volatile amines, widely known as total volatile basic nitrogen (TVB‐N), serves as a robust and universally recognized freshness indicator for protein‐rich foods [55, 126, 127, 128]. While conventional analytical methods for TVB‐N analysis are time‐consuming and require bulky laboratory equipment, modern portable chemiresistors (e.g., poly(3‐hexylthiophene)‐based sensors) can rapidly quantify these volatile amines at the ppb level, effectively assessing meat and fish freshness within minutes [122, 128, 129, 130, 131, 132, 133]. Similarly, terpenes are biologically crucial for agricultural monitoring, acting as primary indicators of plant‐organism communication, environmental stress, and insect infestation [47, 104, 134, 135, 136, 137]. Terpenes exhibit diverse structures and occasionally display chirality owing to their relatively large number of carbon atoms. Humans can discriminate between certain odors of terpene enantiomers through an array of olfactory receptors, which involves chirality and pattern recognition [138, 139]. However, discriminating terpenes via gas sensors is challenging because sensors must recognize slight differences in molecular skeletons based on weak van der Waals interactions between analytes and sensing materials [140, 141, 142]. To systematically address this challenge, rationally designed organic receptors, such as polymers [143], MOFs [141, 144], and peptides [145, 146], have been integrated into advanced sensing platforms. For example, Paolesse et al. synthesized a chiral porphyrin‐based molecule and modified a QCM sensor using the synthesized receptor. The functionalized QCM sensor demonstrated selectivity toward limonene enantiomers, with (*S*)‐(−)‐limonene eliciting a 3.6‐fold higher response than (*R*)‐(+)‐limonene [142].

### 2.3 Environmental Monitoring

VOCs are hazardous air pollutants and toxins that pose substantial risks to human health and the environment. The production of exhaust gases, evaporation of petroleum products, and the use of organic solvents are primary contributors to VOC formation [147]. Aliphatic and aromatic hydrocarbons are significant environment‐related VOCs emitted through various anthropogenic activities, including automobile exhaust, fuel combustion, petroleum refining, and other chemical processes [6, 148]. However, detecting volatile hydrocarbons is challenging because their interactions with sensing materials are limited to weak and nonspecific van der Waals forces, owing to their nonpolar nature. Aromatic hydrocarbons, particularly benzene, toluene, ethylbenzene, and xylene (BTEX), are a critical class of VOCs because of their adverse effects on the environment and human health. Among BTEX, the development of sensitive and selective detection techniques for benzene is of utmost importance because of its toxicity and the challenges associated with its detection. Benzene is classified as a carcinogen across all exposure routes and is linked to leukemia and lymphoma [149]. The Occupational Safety and Health Administration (OSHA, United States) has set an exposure limit for benzene at one part per million (ppm) for an 8‐h work period, highlighting the necessity for achieving ppb‐level detection of benzene [150, 151, 152]. The absence of functional groups and the structural similarity among BTEX compounds complicate the selective detection of benzene despite its high toxicity [153]. Furthermore, benzene's chemical stability hinders chemical reactions on the surface of sensing materials, thereby limiting the sensitivity of benzene detection [150, 154]. Explosive and toxic gases are critical chemical entities in the fields of safety and security [34, 155, 156]. A typical class of explosive VOCs includes nitroaromatic compounds, whose extremely low volatility presents challenges for their detection in gaseous states [157, 158]. Toxic gases often contain sulfur or phosphorus in their chemical structures. Due to their hazardous nature, simulants are commonly used to evaluate the sensing properties of these sensors [159, 160, 161, 162].

## 3 Devices and Their Working Principles for Odor Sensing

In this section, we explore representative VOC sensors and their operational principles. As shown in Table 4, a diverse array of devices and materials has been developed for the detection of VOCs. VOC sensors are classified into three categories based on their output signals: electrical, gravimetric, and optical signals. Electrical sensors generate electrical signals, such as resistance, capacitance, voltage, and current, in response to changes in the chemical environment. These signals are compatible with those of standard electronic systems and can be interpreted as molecular adsorption/desorption events and chemical reactions [163]. Gravimetric sensors typically employ piezoelectric sensing layers, in which measurable shifts in the resonance frequency serve as signals. These frequency shifts were converted into mass changes, indicating the adsorbed amounts and states of VOCs on the sensing surface [164]. Optical sensors, which encompass colorimetric, fluorescent, and plasmonic sensors, utilize changes in the irradiation intensity across the infrared to ultraviolet spectrum, depending on their sensing mechanism. Optical sensors provide multiparametric outputs, such as array‐like color change patterns or spectra, to analyze the chemical interactions between VOCs and sensing materials [165]. Table 5 summarizes a systematic comparison of these representative sensing platforms in terms of system miniaturization, information density, response kinetics, and environmental robustness.

**TABLE 4:** Summary of the sensing devices and materials used for VOC detection.

	C1–C3	C4–C6	C7–C9	C10–
Alcohol/phenol	Chemiresistor (metal oxide [103, 105, 166, 167, 168, 169, 170, 171], thiol‐gold [172, 173], polymer [174, 175, 176, 177, 178], carbon black‐polymer [179], CNT [180, 181, 182, 183, 184], MOF [185], graphene [186, 187, 188], graphene oxide [189], TMD [190, 191, 192], MXene [87, 193, 194]), chemicapacitor (polymer [195, 196], graphene [197], graphene oxide [198], ionic liquid [199, 200], MOF [201, 202], carbon dot [203]), electrochemical (metal oxide [204]), FET (polymer [205], SiNW [206, 207, 208, 209], graphene [210]), QCM (metal oxide [211, 212], organic molecule [213], polymer [214, 215, 216, 217, 218], MOF [219, 220, 221], ionic liquid [222, 223, 224, 225, 226], MXene [227]), SAW (organic molecule [228], graphene oxide [229]), colorimetric (organic molecule [230, 231, 232, 233, 234, 235], MOF [236]), fluorescence (organic molecule [237], MOF [238]), LSPR (thiol‐silver [239], thiol‐gold [240], silica‐gold [241])	Chemiresistor (metal oxide [103, 105, 242], thiol‐gold [243], CNT [180, 181], MXene [244], graphene [188], graphene oxide [189, 245]), chemicapacitor (polymer [196], graphene oxide [198], MOF [202], carbon dot [203]), electrochemical (metal oxide [204]), FET (polymer [205], SiNW [206, 207, 208, 209, 246]), QCM (organic molecule [213, 247], CNT [248], polymer [115, 116, 216, 218, 249, 250], peptide [117], MOF [220, 221, 251, 252, 253], ionic liquid [223, 224], TMD [254]), colorimetric (organic molecule [230, 232, 233, 234, 255, 256], protein [257]), fluorescence (organic molecule [237, 258]), LSPR (silver [259], thiol‐silver [239], thiol‐gold [240], silica‐gold [241]), SERS (graphene oxide‐gold [260])	Chemiresistor (metal oxide [103, 105], thiol‐gold [172, 261], CNT [180, 181], polymer [262], graphene oxide [189, 245, 263]), FET (SiNW [206, 207, 208, 209, 246]), QCM (organic molecule [111, 264], polymer [217], MOF [253], ionic liquid [223]), colorimetric (organic molecule [230, 232, 255, 256, 265, 266, 267], MOF [268]), fluorescence (organic molecule [258]), SERS (LDH‐silver [269], MOF‐gold [270, 271])	Chemiresistor (metal oxide [105], CNT [180]), electrochemical (thiol‐gold [272]), FET (SiNW [206, 207, 209, 246], graphene oxide [273]), QCM (organic molecule [111, 142], polymer [113, 274], MIP [114, 275, 276], MOF [144, 277]), colorimetric (organic molecule [230, 266], MOF [267]), fluorescence (MOF [141], COF [278, 279]), SERS (graphene oxide‐gold [260])
Aldehyde	Chemiresistor (metal oxide [105, 167, 169, 170, 280], polymer [174, 175], MOF [185, 281], TMD [190, 192]), electrochemical (polymer [282], metal oxide [204]), QCM (ionic liquid [283, 284, 285]), colorimetric (organic molecule [108, 233, 286, 287, 288, 289]), fluorescence (SAM [290], MOF [291]), SERS (LDH‐silver [269], MOF‐gold [292], inorganic‐gold [293])	Chemiresistor (metal oxide [103, 105], MOF [185], MXene [87, 294, 295], graphene [188], graphene oxide [189, 245], CNT [296]), chemicapacitor (graphene [197]), electrochemical (polymer [282]), FET (polymer [297], SiNW [246]), QCM (organic molecule [119, 264], polymer [274, 298], MIP [299, 300, 301], graphene oxide [118], TMD [254], ionic liquid [283, 284]), cantilever (polymer [96]), colorimetric (organic molecule [108, 230, 232, 234, 235, 255, 256, 266, 286, 288, 289, 302], MOF [256]), fluorescence (polymer [303], MOF [291]), SERS (LDH‐silver [269], MOF‐gold [270, 292], inorganic‐gold [293])	Chemiresistor (metal oxide [103, 105, 166], MXene [87], polymer [262], ionic liquid [304], graphene oxide [189, 245]), electrochemical (polymer [282], ionic liquid [305]), FET (polymer [297], graphene [210, 306]), QCM (organic molecule [111, 119], MIP [299, 301], graphene oxide [118], ionic liquid [283, 284]), cantilever (polymer [96]), colorimetric (organic molecule [108, 230, 232, 235, 255, 256, 266, 286, 289], MOF [267]), fluorescence (polymer [303], MOF [291, 307], COF [279]), SERS (LDH‐silver [269, 308], inorganic‐gold [293], LDH‐gold [309], MOF‐gold [270, 271, 292], MXene‐gold [310, 311], COF‐gold [312])	Chemiresistor (metal oxide [313, 314], carbon black‐polymer [315], MIP‐gold [316], polymer [262]), electrochemical (polymer [282]), FET (graphene [210, 306]), QCM (polymer [113], MIP [114, 275]), cantilever (polymer [96]), colorimetric (organic molecule [97, 265]), fluorescence (MOF [141]), SERS (MOF‐gold [270, 292])
Ketone	Chemiresistor (metal oxide [76, 167, 169, 170, 171, 317, 318, 319], polymer [174, 175, 176, 177, 178], carbon black‐polymer [179, 315], thiol‐gold [173], CNT [182, 183], graphene [187, 320], graphene oxide [321], MOF [171, 319], MXene [194, 244], TMD [191, 192]), chemicapacitor (polymer [195], ionic liquid [199, 200], MOF [201, 202]), electrochemical (metal oxide [204, 322]), FET (TMD [78]), QCM (metal oxide [211], organic molecule [119, 213], polymer [215, 217], MOF [219], MXene [227], ionic liquid [226, 323]), SAW (organic molecule [228, 324, 325], graphene oxide [229]), colorimetric (organic molecule [231, 233, 234, 286], MOF [236]), fluorescence (organic molecule [326, 327], MOF [238, 328]), LSPR (thiol‐gold [240]), SERS (COF‐gold [312], graphene oxide‐gold [260])	Chemiresistor (metal oxide [242, 318], thiol‐gold [172, 329], polymer [175, 177], CNT [181, 182, 183, 330, 331], graphene [320], graphene oxide [189], MXene [87]), chemicapacitor (graphene [197], ionic liquid [200]), FET (SiNW [209, 246]), QCM (organic molecule [119, 213], ionic liquid [323]), SAW (metal oxide [332]), colorimetric (organic molecule [232, 233, 286], protein [257]), fluorescence (organic molecule [326, 327], polymer [333], MOF [328]), SERS (MXene‐gold [311])	Chemiresistor (metal oxide [103, 166], thiol‐gold [243, 329], MXene [87], ionic liquid [304], graphene oxide [189]), chemicapacitor (polymer [196]), electrochemical (ionic liquid [305]), FET (polymer [297]), QCM (organic molecule [119], ionic liquid [323]), colorimetric (organic molecule [230, 232, 266, 286], MOF [267]), fluorescence (organic molecule [326, 327], polymer [333], MOF [328]), LSPR (thiol‐silver [239], MISG‐gold [334])	Chemiresistor (metal oxide [105], graphene oxide [245], CNT [181], MXene [335], polymer [262]), FET (graphene oxide [273]), QCM (organic molecule [119], MIP [336], MOF [144, 277]), colorimetric (organic molecule [230, 255, 265, 266], MOF [267]), fluorescence (MOF [141], COF [278, 279]), SPR (peptide‐gold [146]), LSPR (MISG‐gold [99])
Carboxylic acid	Chemiresistor (metal oxide [103, 105], polymer [174], carbon black‐polymer [90, 337], graphene [188, 320], graphene oxide [189], MXene [87]), FET (CNT [338], graphene [210, 306]), QCM (metal oxide [212, 339, 340], organic molecule [119], polymer [217], MIP [341]), colorimetric (organic molecule [230, 232, 234])	Chemiresistor (metal oxide [105], carbon black‐polymer [90, 337], graphene [188], graphene oxide [189]), FET (graphene [306], SiNW [342]), QCM (metal oxide [339, 340], organic molecule [119], MIP [341]), colorimetric (organic molecule [230, 232]), LSPR (MISG‐gold [343]), SERS (graphene oxide‐gold [260])	Chemiresistor (graphene oxide [189]), FET (SiNW [342]), QCM (metal oxide [339, 340], MIP [341]), colorimetric (organic molecule [230]), LSPR (MISG‐gold [343])	FET (SiNW [342])
Ester	Chemiresistor (metal oxide [103, 105], polymer [175]), electrochemical (metal oxide [204])	Chemiresistor (metal oxide [103, 105], thiol‐gold [243, 329], polymer [175], carbon black‐polymer [179]), chemicapacitor (polymer [195], ionic liquid [200], carbon dot [203]), QCM (metal oxide [211], organic molecule [213, 344], polymer [250], MOF [253], ionic liquid [285]), colorimetric (organic molecule [232, 233, 235])	Chemiresistor (metal oxide [103, 105], graphene oxide [245, 263]), chemicapacitor (polymer [196]), electrochemical (metal oxide [345], ionic liquid [305]), QCM (organic molecule [111], TMD [254]), colorimetric (organic molecule [230, 232, 235, 255, 265, 266], MOF [256, 267]), LSPR (thiol‐silver [239], gold [346]), SERS (graphene oxide‐gold [260])	Chemiresistor (metal oxide [105, 166], graphene oxide [245], MXene [335]), QCM (MIP [347]), cantilever (polymer [96]), colorimetric (organic molecule [255, 266], MOF [267]), fluorescence (MOF [141])
Amine	Chemiresistor (metal oxide [348], thiol‐gold [172], polymer [128, 174, 177], carbon black‐polymer [337], MOF [349], COF [350], MXene [351]), chemicapacitor (metal oxide [352], polymer [122, 196]), FET (organic molecule [353]), QCM (metal oxide [212], polymer [354], COF [355], ionic liquid [283]), colorimetric (organic molecule [132, 287, 356, 357, 358, 359]), fluorescence (organic molecule [129, 131, 360], polymer [130, 361], MOF [291], COF [362])	Chemiresistor (metal oxide [363, 364], carbon black‐polymer [337], CNT [133, 183], MOF [185, 349, 365], graphene [186, 188], MXene [244, 294, 366, 367]), chemicapacitor (polymer [122]), FET (organic molecule [353]), QCM (metal oxide [212], organic molecule [142, 214, 247, 368, 369], polymer [354], peptide [370], COF [355]), colorimetric (organic molecule [132, 231, 232, 356, 371]), fluorescence (organic molecule [129, 131], polymer [130, 361, 372], MOF [291], COF [362]), SERS (COF‐gold [312])	QCM (organic molecule [373, 374], MOF [253]), colorimetric (organic molecule [230, 232, 356, 371]), fluorescence (polymer [361, 372]), SERS (MOF‐gold [375])	QCM (organic molecule [142]), colorimetric (organic molecule [230])
Aliphatic hydrocarbon	Chemiresistor (metal oxide [169, 170, 376, 377, 378], MOF [379], CNT [380]), FET (CNT [109]), colorimetric (organic molecule [359]), fluorescence (polymer [381])	Chemiresistor (metal oxide [72, 73, 74, 75, 103, 167, 382, 383, 384], thiol‐gold [243], polymer [174, 175, 176, 177, 178], carbon black‐polymer [179], CNT [180, 183, 184, 385], MOF [185, 379], graphene [187, 188, 320], graphene oxide [189, 321], TMD [190, 191, 192], ionic liquid [304], MXene [194]), chemicapacitor (graphene oxide [198], ionic liquid [200], MOF [202], carbon dot [203]), electrochemical (metal oxide [322], ionic liquid [386], gold [387], graphene oxide [388]), FET (polymer [389], SiNW [206, 207, 208, 209, 246]), QCM (metal oxide [212], organic molecule [247], polymer [390], MOF [220, 221, 251], ionic liquid [222, 285]), SAW (organic molecule [228, 325], graphene oxide [229]), colorimetric (organic molecule [230, 234, 302, 391], MOF [236]), fluorescence (organic molecule [392, 393, 394], polymer [381], COF [395], MOF [238]), LSPR (thiol‐gold [240]), SERS (LDH‐gold [309], graphene oxide‐gold [260])	Chemiresistor (metal oxide [103, 318], thiol‐gold [261, 329], CNT [180, 181, 184, 385], MOF [185], graphene [187], graphene oxide [189]), chemicapacitor (polymer [195], graphene [197]), electrochemical (metal oxide [322], ionic liquid [386], gold [387], graphene oxide [388]), FET (polymer [389], SiNW [206, 207, 208, 209, 246]), QCM (metal oxide [212], polymer [390], ionic liquid [222, 225]), SAW (polymer [396, 397, 398], metal oxide [332]), colorimetric (organic molecule [230, 234]), fluorescence (organic molecule [393], polymer [381]), LSPR (silver [259], thiol‐silver [239]), SERS (graphene oxide‐gold [260])	Chemiresistor (CNT [180, 181, 184, 385], thiol‐gold [329]), FET (polymer [389], SiNW [206, 207, 208, 209, 246]), QCM (polymer [390]), colorimetric (organic molecule [230, 234]), fluorescence (organic molecule [393], polymer [381]), SERS (graphene oxide‐gold [260])
Aromatic hydrocarbon		Chemiresistor (metal oxide [149, 150, 154, 170, 318, 399, 400, 401, 402, 403, 404, 405, 406], thiol‐gold [173, 329, 407], polymer [174, 176, 178], carbon black‐polymer [179, 408], CNT [181, 183, 409, 410, 411, 412], MOF [185, 413], MXene [244], graphene [187, 188], TMD [191], ionic liquid [304]), chemicapacitor (polymer [195, 414], MOF [201], COF [415]), electrochemical (metal oxide [322]), FET (polymer [389], CNT [338, 416, 417]), QCM (metal oxide [212], organic molecule [213], polymer [216, 274, 418], CNT [412], MOF [219, 221, 419, 420], ionic liquid [222, 225]), SAW (organic molecule [325]), cantilever (MOF [421]), colorimetric (organic molecule [230, 231, 232, 233, 234]), fluorescence (polymer [422, 423], MOF [238]), LSPR (silica‐gold [241]), SERS (MOF‐silver [424], inorganic‐gold [425], LDH‐gold [309], MOF‐gold [426], MXene‐gold [311])	Chemiresistor (metal oxide [150, 154, 167, 169, 317, 318, 348, 382, 399, 400, 401, 402, 404, 427, 428, 429], thiol‐gold [172, 243, 261, 329, 407, 430], polymer [174, 176, 177, 178, 412], carbon black‐polymer [179, 408], CNT [181, 183, 385, 409, 411, 431], MOF [185], graphene [187, 320], graphene oxide [189], TMD [190, 191, 192], MXene [87, 193, 194, 244, 432], ionic liquid [304]), chemicapacitor (polymer [195, 414], graphene [197], graphene oxide [198], ionic liquid [200], carbon dot [203]), electrochemical (metal oxide [204, 322]), FET (polymer [297, 389, 433], CNT [416, 434], SiNW [209]), QCM (metal oxide [211, 212], organic molecule [213, 247, 435], polymer [216, 217, 249, 274, 412, 418], MIP [436], CNT [431], MOF [219, 221, 419, 420, 437], ionic liquid [222, 226]), SAW (metal oxide [332, 398], organic molecule [228, 324, 325, 438], polymer [396, 397], graphene oxide [229]), cantilever (MOF [421]), colorimetric (organic molecule [230, 232, 233, 234, 302], MOF [236]), fluorescence (polymer [422, 423, 439], MOF [238]), LSPR (silver [259], thiol‐silver [239], thiol‐gold [240], silica‐gold [241]), SERS (MOF‐silver [424], inorganic‐gold [425], LDH‐gold [309], MOF‐gold [440])	Chemiresistor (polymer [174], CNT [180], graphene [187], thiol‐gold [329]), FET (CNT [338])
Terpene/terpenoid				Chemiresistor (metal oxide [71, 105], polymer [143], MXene [335], thiol‐gold [329]), electrochemical (thiol‐gold [441], graphene oxide [388]), FET (graphene [140, 145], CNT [442]), QCM (organic molecule [142, 344, 374, 443, 444], polymer [113], MIP [114, 276, 445], MOF [144, 253, 277, 446, 447], TMD [254]), colorimetric (organic molecule [233, 235]), fluorescence (MOF [141], COF [278]), SPR (peptide‐gold [146]), LSPR (MISG‐gold [99, 334])

**TABLE 5:** Summary table of VOC sensing devices.

Sensing devices	System miniaturization & scalability	Information density	Response & recovery kinetics	Environmental robustness (e.g., humidity)
Chemiresistors	High	Low	Moderate	Low
Chemicapacitors	High	Low	Moderate	Low
Electrochemical sensors	Moderate	Low–moderate	Fast	Moderate
FETs	High	Moderate	Fast	Low
QCM sensors	Moderate	Low	Moderate	Moderate
SAW sensors	Moderate	Low	Fast	Low
Colorimetric sensors	Moderate	Moderate	Slow–moderate	Low
Fluorescent sensors	Low	High	Fast	Moderate
Plasmonic sensors	Low	High	Fast	Moderate

### 3.1 Electrical Sensors

#### 3.1.1 Chemiresistors

Chemiresistors are sensors characterized by an intrinsic baseline resistance that responds to changes in the chemical environment, such as exposure to gaseous analytes. These sensors have been employed in a diverse array of applications owing to their numerous advantages such as ease of fabrication, low‐power operation, straightforward output, and sensitivity to gases [448, 449, 450]. However, because their output is a single parameter (resistance/conductance), chemiresistors are often integrated into sensor arrays. A variety of materials have been utilized as sensing materials in chemiresistors, including metal oxides [166, 451], metal nanoparticles [172, 243, 452], polymers [174, 175], CNTs [180, 181, 182, 385], and 2D conductive materials [185, 190, 193, 321]. Although various materials can be employed as chemiresistors, the mechanisms by which they modulate resistance differ, and the selection of chemiresistive materials influences the characteristics of the extracted VOC analytes. Metal oxide semiconductors typically function at elevated temperatures, where weak physical adsorption is thermodynamically unfavorable because of rapid desorption. Instead, the response is dominated by chemisorption, involving redox reactions with surface oxygen ions, which complicates the extraction of noncovalent interactions, despite their advantage in large‐scale integration [453, 454]. Two‐dimensional materials, such as graphene, transition metal dichalcogenides (TMDs), and MXenes, facilitate room‐temperature operation, thereby extending the lifetime of the physically adsorbed molecules. However, their signal transduction is primarily driven by charge transfer, which may obscure the differences in molecular structures [455, 456]. In contrast, polymer‐based composites and metal nanoparticle networks inherently operate at room temperature and transduce signals based on the thermodynamic partition equilibrium between the VOC and the soft organic matrix or ligand [457, 458]. In polymer composites, the chemical affinity of VOCs drives their absorption into the polymer matrix. This absorption induces volumetric swelling that significantly disrupts the percolating network of conductive fillers, thereby modulating the macroscopic resistance [459]. Similarly, in metal nanoparticle networks, such as thiol‐capped gold, VOCs partition into the organic ligand shell. This partitioning induces swelling that exponentially increases the interparticle electron tunneling resistance and concurrently alters the local dielectric environment [172]. Consequently, these organic–inorganic hybrid platforms are thermodynamically suitable for extracting subtle physical adsorption characteristics essential for smelltronics. Capitalizing on this mechanism, Peng et al. decorated gold nanoparticles with organic molecules containing thiol groups, fabricated chemiresistors based on these functionalized nanoparticles, and integrated them into a nine‐channel array. The interactions between VOCs and nanoparticles modulate the interparticle distances, leading to changes in the resistance of chemiresistors. This cross‐reactive sensor array was capable of distinguishing between patients with lung cancer and healthy controls based on their breath, without the need for dehumidifying or pre‐concentrating exhaled biomarkers [66]. Although organic‐based chemiresistors are proficient in extracting thermodynamic partitioning, integrating a substantial number of these sensors presents a considerable fabrication challenge. To advance the development of smelltronics through an alternative methodology—namely, the acquisition of extensive, high‐dimensional data for artificial intelligence (AI) analysis—researchers have extensively utilized the scalability of metal oxide semiconductors [453]. Honda et al. successfully integrated 1024 nanofilm channel SnO_2_ sensors into a single chip to develop a reliable sensing system. Lateral nanofilm channel devices combined with crossbar interconnect geometry were employed to mitigate interconnect parasitic resistance, thereby enabling robust and rapid sensing of VOCs by the highly integrated chemiresistor array chip [460].Taking integration to an even higher level, Wang et al. developed a highly integrated chemiresistor array on nanotubes, where each chip employed 10 000 distinct sensors, despite the fact that the sensor array comprised only a few metal oxides. They fabricated SnO_2_‐based chemiresistive sensing layers on nanotubes and subsequently applied a multicomponent interfacial layer between the sensing layer and the top electrodes (Figure 3a). This multicomponent interfacial layer comprises four metal oxides (ZnO, NiO, In_2_O_3_, and WO_3_) with varying gradient distributions, resulting in numerous compositions with distinct properties. It modulated the carrier transport between the top electrode and sensing layer, resulting in a sensor array with a large number of sensing elements, even though it contained only four metal oxide materials (Figure 3b). The sensor array exhibited high sensitivity to various gases, with the ability to distinguish between mixed gases and odors (Figure 3c,e) [167]. As an alternative to such massive spatial integration, Tang et al. discriminated VOCs using a single chemiresistor by applying pulsed heat. They fabricated a Pd‐decorated SnO_2_ chemiresistor and modulated its temperature by applying a pulsed voltage. Pulse heating varied the carrier concentrations and surface reactivity of the chemiresistor on a short timescale, enabling the extraction of characteristic diffusion and reaction kinetics of VOCs on the sensing surface, which are advantageous for molecular discrimination [317]. Despite the implementation of advanced integration and modulation strategies, chemiresistive platforms inherently encounter challenges in terms of environmental robustness. Their straightforward electrical outputs are notably vulnerable to ambient humidity and long‐term baseline drift, necessitating meticulous system calibration to ensure reliable odor recognition in real‐world applications.

**FIGURE 3:**
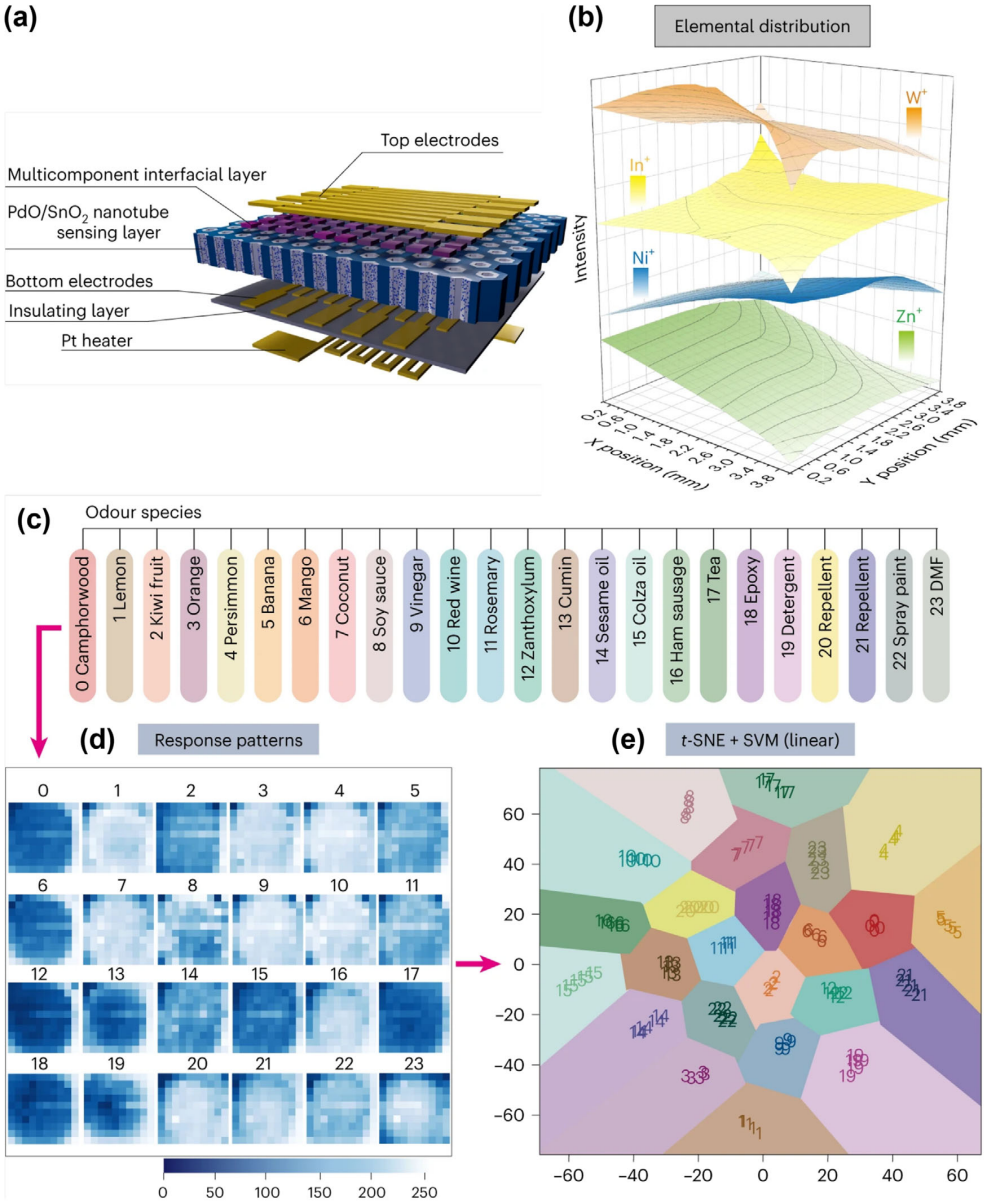
(a) A schematic representation of the monolithic chemiresistor array is presented, featuring a multicomponent interfacial layer designed to induce subtle variations in the sensing properties of the device. (b) The spatial distribution of elements within the multicomponent interfacial layer was visualized using time‐of‐flight secondary ion mass spectrometry and x‐ray diffraction. (c) VOCs and odors selected for detection using the chemiresistor array were identified. (d) Normalized response patterns for the targeted VOCs and odors are shown. (e) VOCs and odors were classified using *t*‐distributed stochastic neighbor embedding (*t*‐SNE) and support vector machine (SVM) with a linear kernel algorithm. Reproduced with permission [167]. Copyright 2024, Springer Nature.

#### 3.1.2 Chemicapacitors

Chemicapacitors exhibit variable capacitance, which is indicative of changes in the permittivity of the dielectric layer. Upon exposure to VOCs, the dielectric layer of the chemicapacitor absorbs these compounds and induces changes in the dielectric properties and/or thickness, resulting in a change in capacitance. Chemicapacitors are characterized by their simple structures and ability to function without the need for heating, mechanical excitation, or light sources, rendering them cost‐effective and energy‐efficient sensing platforms. However, the interactions between dielectric materials and sensing analytes at room temperature can impede molecular diffusion, thereby delaying both the response and recovery processes [195]. The selectivity of chemicapacitors is contingent upon the interactions between dielectrics and analytes, prompting the use of diverse materials such as polymers [196, 414, 461], graphene [197, 198], ionic liquids (ILs) [199, 200], MOFs [201, 202, 420, 462, 463], and other materials [352, 415, 464]. These materials have been employed as dielectrics to design chemicapacitive sensing devices. The operation of chemicapacitors at room temperature can be advantageous for low‐power applications and molecular selectivity by facilitating the extraction of weak noncovalent interactions (e.g., van der Waals interactions) between analytes and sensing materials [465, 466]. Capman et al. developed a sensor array composed of graphene‐based chemicapacitors. Thirty‐six distinct organic molecules, including cavitands and metalloporphyrins, were used to functionalize graphene‐based chemicapacitors, which were subsequently integrated into a sensor array. This graphene‐based sensor array demonstrated high accuracy in discriminating VOCs, achieving a 75% accuracy rate in distinguishing between octane and 1‐octene despite their chemical similarity [197]. Shauloff et al. synthesized carbon dots with varying polarities and applied them to interdigitated electrodes to construct a chemicapacitor array. This array exhibited cross‐reactivity based on polarity matching between the carbon dots and VOC analytes. The sensor array was further tested with VOC mixtures emitted from gram‐negative (*E. coli* and *P. aeruginosa*) and gram‐positive bacteria (*B. subtilis* and *S. aureus*), successfully discriminating each species [203]

#### 3.1.3 Electrochemical Sensors

Electrochemical sensors detect VOCs through redox reactions that produce measurable signals, such as current, potential, or changes in conductivity, which correlate with analyte concentrations. Redox reactions of VOCs occur at the working and counter electrodes, where the generated chemical species and carriers are transported through the electrolyte interlayer [60, 448, 467]. These sensors can differentiate VOCs based on their intrinsic redox potentials and have been employed to detect VOCs in breath, including straight‐chain aldehydes [282], aliphatic/aromatic hydrocarbons [322, 386], isoprene [387], limonene [441], and other volatile biomarkers [204, 272, 305, 345, 388], to facilitate noninvasive disease diagnosis. Obermeier et al. developed an electrochemical sensor for straight‐chain aldehydes, which was tested on breath samples from patients with lung cancer, smokers, and healthy controls, demonstrating its capability to detect aldehydes at low ppb levels [282]. Nazir et al. enhanced the electrodes of electrochemical sensors using hexanethiol‐capped Au nanoparticles. The interactions between the thiolated capping agent and limonene vapors improved the sensitivity, enabling the quantification of limonene in breath samples from cirrhotic patients [441]. Although electrochemical sensors are regarded as among the most promising types of wearable VOC sensors owing to their high quantifiability, research on detecting VOCs in the gas phase is predominantly limited to highly volatile or polar molecules [60, 468, 469]. These sensors operate based on the faradaic redox reactions of VOCs, which are significantly influenced by the electroactive functional groups of the analytes and their concentrations, thereby emphasizing the challenges associated with low‐reactive and low‐volatile molecules. Moreover, the inherent dependence on liquid, gel, or solid electrolytes presents considerable challenges in terms of long‐term environmental durability and sensor longevity. Prolonged exposure to ambient conditions frequently results in electrolyte evaporation, leakage, or gradual degradation, which significantly undermines baseline stability.

#### 3.1.4 FETs

A FET modulates carrier transport between the source and drain electrodes by applying a gate voltage, thereby regulating the conductivity of the semiconductor channel. Compared to conventional two‐terminal sensors, FETs offer distinct advantages for artificial olfactory systems, including miniaturization, low power consumption, and the ability to extract multiple independent parameters (e.g., threshold voltage and mobility) by leveraging the controllability of the gate voltage. In addition, the channel conductance varies based on the interaction with VOCs, facilitating their detection and quantification. Mechanistically, the dominant sensing mechanism of FET‐based devices depends on the polarity of analytes and can be categorized into three types: (i) electron transfer between channels and analytes, (ii) changes in the work function of gate metal electrodes caused by analyte adsorption, and (iii) the effect of analyte adsorption on the surface scattering and trapping of charge carriers [470, 471]. Owing to the intrinsic signal amplification provided by the transistor architecture, these subtle interfacial interactions are effectively translated into significant modulations of the drain current, making FETs highly sensitive platforms for capturing complex odor information [448, 472]. Various materials, including polymers [205, 297, 389, 433], graphene [140, 306, 473], CNTs [338, 442], and silicon nanostructures [206, 207, 208, 246, 342, 474], have been functionalized for use in FET applications. Yamazaki et al. functionalized graphene with peptides to fabricate FETs, in which peptides were noncovalently immobilized and formed molecular films through self‐assembly on the graphene. FETs with peptide receptors demonstrated enantioselective sensor responses to limonene, detecting (R)‐(+)‐limonene with a 35‐fold higher response than (*S*)‐(−)‐limonene under humid conditions. Importantly, although ambient moisture is generally regarded as a detrimental interference in gas sensing, in this context, it functions as a structural facilitator. Water molecules absorbed by the peptide layer, as indicated by humidity‐induced swelling, confer essential conformational flexibility to the receptors. This moisture‐induced plasticity allows the peptides to dynamically adjust and capture specific limonene enantiomers in their more energetically favorable and stereospecific conformations, thereby markedly enhancing enantioselectivity [140]. While functionalizing the sensing layers enhances selectivity, relying solely on massive sensor arrays for molecular discrimination often introduces significant integration challenges. Furthermore, such highly integrated arrays often lack long‐term stability owing to drift or aging of the sensing materials, which is uncorrelated among different sensors [475]. To circumvent these bottlenecks, FETs are promising platforms for acquiring high‐dimensional data because a single FET sensor can output multiple signals, and the resulting signal patterns can be used to discriminate between different VOCs. Wang et al. achieved VOC discrimination using a single FET sensor composed of silicon nanowires (SiNWs) functionalized with self‐assembled monolayers (SAMs). They extracted four independent parameters from a single FET sensor: threshold voltage, hole mobility, subthreshold swing, and source‐drain current at a specific gate voltage. These parameters were used as inputs for the artificial neural network (ANN) models. Proper modification of the SiNWs with SAMs and trained ANN models enabled the discrimination of similar VOCs, such as alkanes (hexane, octane, and decane) and alcohols (ethanol, hexanol, octanol, and decanol), using a single FET device [209]. The primary challenge associated with FET‐based VOC sensors is their robustness. This is due to the fact that the charge transfer and sensing characteristics of FETs are significantly influenced by factors such as gate bias, trapped charges or ionized molecules in proximity to the sensing channel, and humidity [476].

### 3.2 Gravimetric Sensors

Gravimetric sensors, including QCM, SAW, film bulk acoustic resonator (FBAR), and cantilever, enable VOC sensing based on the direct conversion of the analyte adsorption/desorption events into measurable frequencies. Therefore, a wide range of well‐explored materials with adsorption capabilities can be utilized to functionalize gravimetric sensors [164]. Fundamentally, the sensing mechanism of acoustic resonators is traditionally described by the linear mass‐loading effect (e.g., the Sauerbrey equation for QCM), which establishes a linear relationship between the mass of the adsorbed molecules (Δ*m*) and the resulting shift in the resonance frequency (Δ*f*), assuming that the sensing layer functions as a rigid, thin film [477]. However, when soft organic matrices are used to capture large VOCs, this rigid‐film approximation fails because of viscoelastic softening and energy dissipation. Therefore, the adhesion between the sensing materials and the resonator electrodes should be carefully engineered [370, 478, 479].

#### 3.2.1 QCM Sensors

QCM sensors are piezoelectric devices that quantify the mass of molecules adsorbed or desorbed from surfaces using bulk acoustic waves. The change in mass owing to molecular adsorption and desorption on the QCM surface results in a characteristic shift in the resonant frequency, as described by the Sauerbrey equation. The sensing material applied to the electrode of a QCM sensor is crucial for determining its sensing characteristics, given that QCM sensors are mass‐sensitive but lack specificity [477, 480]. A variety of functional materials, such as polymers [249, 274, 275, 481], metals/metal oxides [211, 212, 482], and MOFs [219, 220, 251, 419, 420, 437], have been used to enhance the sensing capabilities of QCM sensors. QCM sensors with diverse surface modifications have been incorporated into sensor arrays to differentiate between analytes [214, 222, 336, 390]. For example, a QCM sensor array modified with MOFs can distinguish between *o*‐, *m*‐, and *p*‐xylenes and their ternary isomer mixtures. The distinct pore sizes of each MOF contributed to its unique molecular adsorption characteristics, thereby facilitating molecular discrimination based on cross‐reactivity (Figure 4a) [437]. The high‐quality factors (Q) of acoustic resonators are critical for detecting VOCs with high sensitivity. A lower quality factor diminishes the detectability of these devices because of increased noise. Consequently, it is imperative to meticulously regulate sensitizing materials, particularly organic materials with low adhesion, to optimize their positive impact on the sensing properties while mitigating their detrimental effects on the quality factor [164, 370, 479, 483, 484, 485]. Khoshaman and Bahreyni applied MOF (HKUST‐1) films to QCM sensors using drop‐casting and electrospraying methods. The conventional drop‐cast MOF‐coated QCM sensor exhibited a noisy and irreversible response to isopropanol, with a quality factor of 850. In contrast, the electrosprayed MOF‐coated QCM sensor exhibited reversible signals with reduced noise, and its quality factor was calculated to be 5000. The variations in the quality factors and sensing performances of the QCM sensors were attributed to the uniformity of the MOF films, underscoring the importance of investigating the growth methodology of functional materials on gravimetric devices [479]. Reducing the number of sensor components or materials while preserving their discriminatory capabilities is essential for the straightforward fabrication and long‐term stability of practical sensors. Qin et al. developed a QCM sensor array using a single MOF material whose adsorption properties were incrementally modified through light irradiation. They synthesized MOF films incorporating photoresponsive azobenzene side groups, whose cis–trans conformations could be adjusted by varying the light wavelength. Identical MOF films were applied to four QCM sensors to form a sensor array, and their structures and adsorption properties were modulated by light irradiation (Figure 4b). The performance of the 4‐channel QCM sensor array in detecting and classifying VOCs was suboptimal when all four sensors shared the same configuration. Conversely, light irradiation induced different cis–trans conformations in the MOF films, resulting in four distinct QCM sensors with cross‐reactivity, thereby enhancing classification accuracy (Figure 4c) [220]. Virtual sensor array methodologies can reduce the number of sensor components by deriving multiple parameters from single sensors. Capitalizing on these viscoelastic properties, techniques such as QCM with dissipation monitoring (QCM‐D) [223, 224, 486, 487] and the Butterworth–Van Dyke equivalent model [215, 227] have been explored, demonstrating their ability to discriminate VOCs by extracting multiple sensing parameters from a single sensor. Speller et al. employed a QCM‐D virtual sensor array to classify VOCs using a single sensor. They coated a QCM‐D sensor with ILs and recorded the frequency shifts when exposed to VOCs at various harmonics. A single QCM‐D sensor produced multiple frequency shifts corresponding to harmonic numbers, resulting in unique response patterns for VOC discrimination [223]. Although these innovative strategies aim to optimize the information output from individual devices, QCM platforms inherently face challenges related to system scalability. The unavoidable dependence on relatively large oscillator circuits significantly limits their integration into dense cross‐reactive arrays, which are envisaged for portable sensing systems.

**FIGURE 4:**
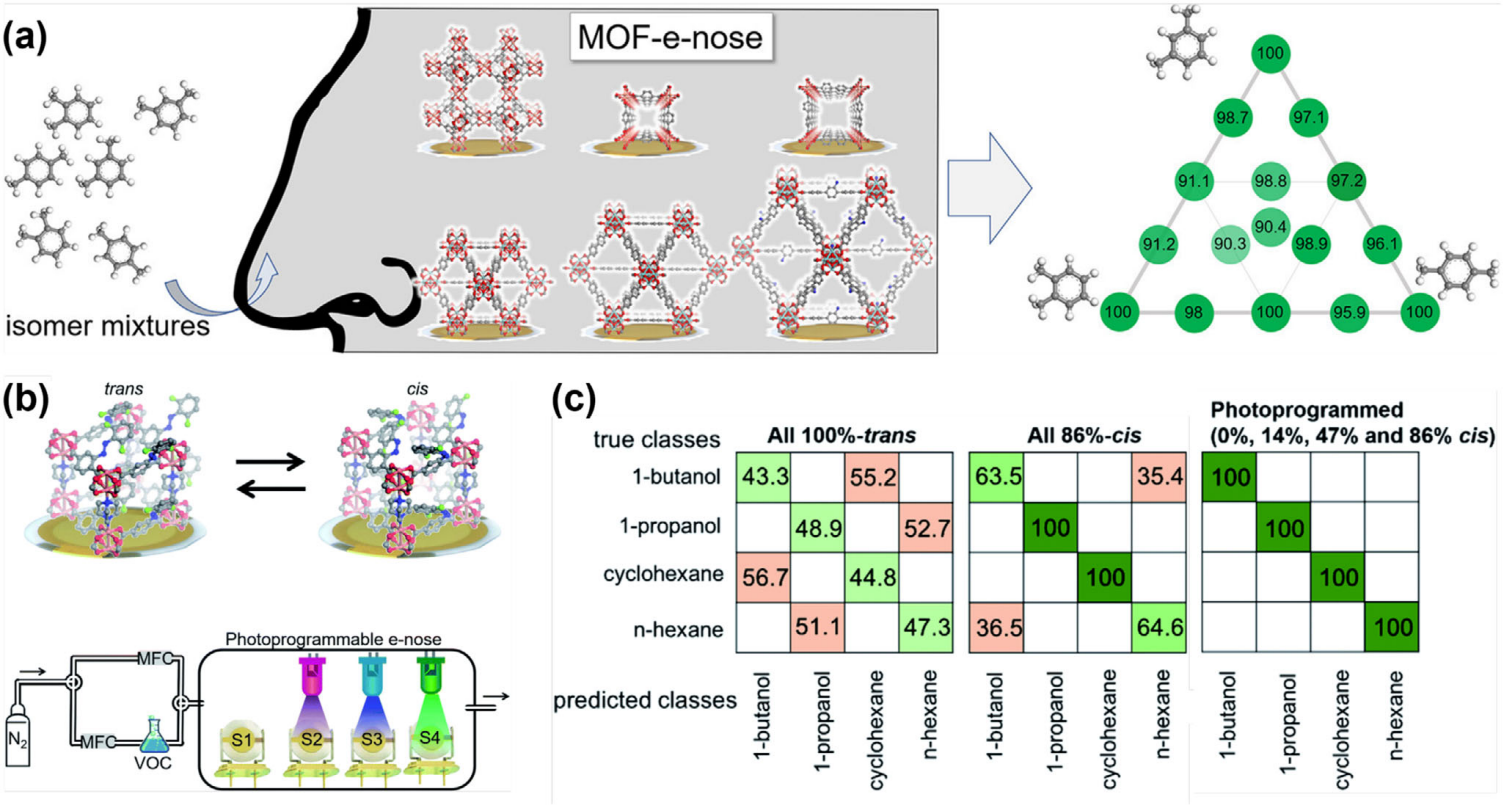
(a) MOF‐based QCM sensor array designed for the differentiation of *o*‐, *m*‐, and *p*‐xylene, as well as their ternary mixtures. Reproduced with permission [437]. Copyright 2022, American Chemical Society. (b) Depictions of photoresponsive MOFs on a QCM sensor, where azobenzene side groups can modulate their *cis*‐*trans* conformations. The illustration also includes the concept of an MOF‐based QCM sensor array comprising a single receptor and VOC‐sensing configuration. (c) A confusion matrix illustrating the classification of VOCs using the k‐nearest neighbor (kNN) machine learning algorithm. The average accuracy for VOC classification was notably low when the MOFs were in uniform states, specifically in the all‐100%‐*trans* or all‐86%‐*cis* states. Photoprogramming the conformations of MOFs induces cross‐reactivity, thereby enhancing classification accuracy. Reproduced with permission [220]. Copyright 2021, Royal Society of Chemistry.

#### 3.2.2 SAW Sensors

Although QCMs excel at extracting fundamental thermodynamic and viscoelastic parameters, their bulk acoustic wave configuration and sandwich‐electrode structure fundamentally limit their scalability for massive array integration. SAW sensors are gravimetric sensors that detect mass changes by propagating acoustic waves along the surfaces of the devices. The characteristics of this acoustic wave, such as the velocity or amplitude, are altered by interactions with analytes, leading to observable shifts in the frequency or phase [164, 488, 489]. Owing to their higher resonance frequencies, SAW sensors typically exhibit more pronounced responses compared to QCM sensors [229]. Various materials, including polymers [332, 398, 490, 491], cavitands [228, 324, 325, 438], CNTs [396, 397], and others [229, 492], have been used to modify the sensing properties of SAW sensors. In a study by Chen et al., SAW sensors were integrated with solid‐phase microextraction (SPME) and gas chromatography (GC) techniques to detect lung cancer by analyzing breath samples. The SAW sensor was coated with poly(isobutylene) and used as a VOC detector. VOCs in the breath samples were pre‐concentrated using SPME and separated by GC before being transported to the SAW detector. A single polymer‐coated SAW sensor was able to detect VOCs and generate response patterns based on the retention time of each analyte, effectively distinguishing lung cancer patients from healthy controls [490]. Although SAW sensors facilitate cost‐effective fabrication and low‐power operation, they are susceptible to environmental fluctuations such as variations in ambient temperature and humidity. Additionally, the integration of multiple SAW devices into cross‐reactive arrays is significantly impeded by crosstalk between adjacent high‐frequency oscillator circuits.

### 3.3 Optical Sensors

#### 3.3.1 Colorimetric Sensors

In the field of smelltronics, colorimetric sensor arrays signify a significant paradigm shift from the measurement of specific parameters to high‐dimensional optical pattern recognition. These arrays serve as effective platforms for practical VOC‐sensing applications because they can display distinct color‐change patterns that are readily discernible by humans, machine vision systems, and commonly used devices, such as smartphones. While gravimetric and certain chemiresistive platforms primarily focus on extracting physical partitioning parameters, colorimetric sensor arrays utilize a strategically curated library of chemoresponsive dyes to explore a wide range of chemical reactivities and polarities. These arrays incorporate Brønsted acidic or basic dyes, Lewis acid/base dyes, redox dyes, colorants with substantial permanent dipoles, and chromogenic aggregative materials [493]. By exploiting this diverse cross‐reactivity rather than relying solely on weak and nonspecific van der Waals interactions, colorimetric sensor arrays translate complex molecular interactions into a combinatorial spatial map of optical signals (e.g., ΔR, ΔG, and ΔB). Consequently, despite the irreversibility of color changes, these arrays are particularly adept at detecting and differentiating coordinating VOCs, such as amines, thiols, and acids [230]. Rakow and Suslick pioneered the concept of an optoelectronic nose by developing a cross‐reactive colorimetric sensor array utilizing metalloporphyrins to discriminate coordinating VOCs through Lewis acid–base interactions. They immobilized tetraphenylporphyrins with various metal centers on reversed‐phase silica thin‐layer chromatography (TLC) plates. Each metalloporphyrin component exhibited distinct chemical hardness and ligand‐binding affinity, resulting in diverse color‐change patterns upon exposure to VOCs [231]. Since this development, colorimetric sensor arrays have been extensively studied to explore their practical applications and enhance their sensing performance. These arrays have demonstrated their ability to distinguish beverages [232, 286, 494, 495], perfumes [265, 268], food ripening/spoilage [108, 236, 257, 358], bacteria [496], and toxic gases, based on the VOCs emitted from these analytes [287, 497]. Li et al. developed a colorimetric sensor array to discriminate volatile aldehydes and ketones at the ppm and sub‐ppm levels using amine‐containing indicators (Figure 5a). Given that aldehydes and ketones significantly contribute to the aroma of many beverages, these VOCs are considered to be meaningful analytes. The colorimetric sensor array successfully distinguished volatile aldehydes and ketones at ppb levels and generated distinctive color patterns when exposed to liquors (Figure 5b) [286]. Wang et al. enhanced the sensitivity and humidity resistance of colorimetric sensors by combining dyes with MOFs and COFs. The dyes were adsorbed onto the MOFs and subsequently covered with hydrophobic COFs shells. The MOFs uniformly distributed the dyes and improved the sensitivity toward VOCs, whereas the hydrophobic COF shells grown on the MOFs mitigated humidity interference. These features facilitate the accurate and early detection of plant diseases based on the emitted VOCs [266]. High‐dimensional optical data matrices produced by diverse colorimetric sensor arrays serve as optimal inputs for sophisticated ML algorithms. This facilitates the quantitative interpretation of subtle cross‐reactive color variations into precise odor classification. However, these colorimetric sensors face inherent trade‐offs related to continuous monitoring and miniaturization. The robust chemical interactions responsible for color shifts are often irreversible and prone to photodegradation, limiting colorimetric sensors to single‐use, disposable applications, rather than enabling real‐time dynamic recovery. Additionally, the strict requirements for external imaging hardware and stable illumination conditions pose significant physical challenges for their integration into compact and continuously operating sensing platforms.

**FIGURE 5:**
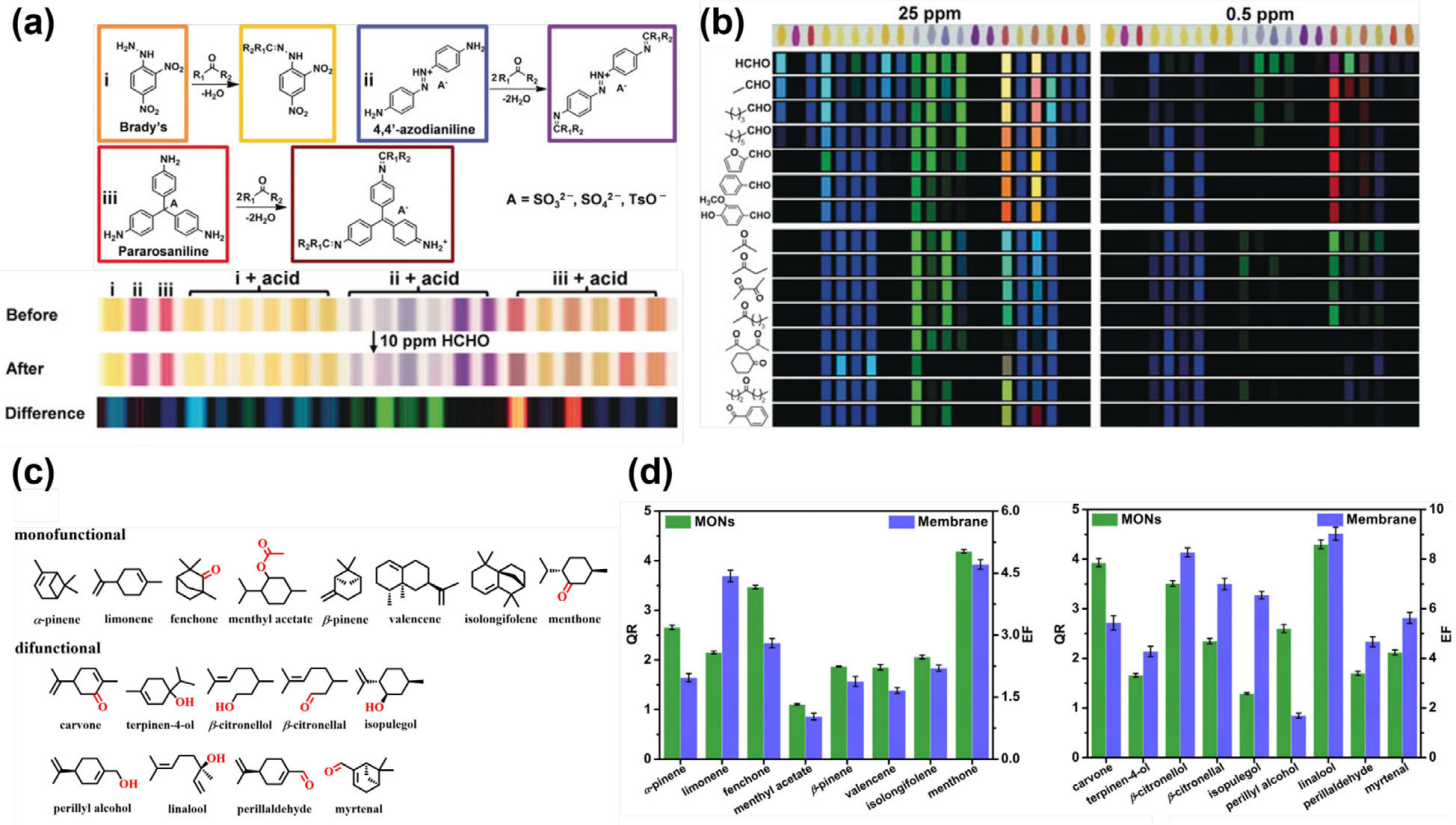
(a) Dyes used in the aldehyde/ketone‐responsive colorimetric sensor array, illustrating the color variations before and after exposure to VOCs. (b) Color difference profiles for seven aldehydes and eight ketones at concentrations of 25 and 0.5 ppm. Reproduced with permission [286]. Copyright 2017, Wiley. (c) Chemical structures of chiral volatile terpenes and terpenoids examined in this study. (d) Quenching ratio (QR) and enantioselectivity factor (EF) of the fluorescence of the MOF nanosheets for the 17 terpenes and terpenoids. Reproduced with permission [141]. Copyright 2021, American Chemical Society.

#### 3.3.2 Fluorescent Sensors

Fluorescent sensors serve as highly sensitive optical transducers, enabling the visualization of local thermodynamic and structural changes within receptor matrices upon VOC exposure. The interactions between fluorescent probes and target VOCs induce perturbations in the local microenvironment, thereby affecting parameters such as polarity, electron/energy transfer, and molecular conformation. These perturbations directly result in measurable changes in the fluorescence intensity or wavelength. Various fluorescent materials, including films of organic molecules [237, 258, 326, 327, 392, 393], polymers [130, 303, 333, 361, 372, 381, 422, 423, 439], COFs [278, 362], and MOFs [141, 291, 307, 328] have been explored for VOC sensing. Through these noncovalent physical interactions, fluorescent probes can distinguish minute differences in molecular structures, such as carbon skeletons or chirality, which are critical and challenging for the practical application of VOC sensors. Li et al. successfully differentiated the alkyl chain lengths of aliphatic ketones using pillararene‐based fluorescent crystals. The as‐synthesized pillararene crystals exhibited yellow fluorescence, which was attributed to the *H‐aggregation* of anthracene excimers. When exposed to 2‐butanone, 2‐pentanone, and 2‐hexanone, the pillararene crystals exhibited a fluorescence shift to green, whereas they retained a yellow fluorescence when exposed to heptanone and octanone. The uptake of ketones with shorter alkyl chains in the interspaces between pillararenes induces the disassembly of anthracene excimers into *J*‐aggregated monomers, resulting in a blue‐shifted green emission. In contrast, heptanone and octanone were unable to occupy the space between pillararenes because of their larger molecular volumes, demonstrating a size‐exclusion mechanism governed by physical partitioning [327]. Liu et al. synthesized chiral fluorescent MOFs and exfoliated them to produce single‐crystalline two‐dimensional nanosheets. The molecular recognition of the MOF nanosheets was analyzed using vapors of 17 chiral terpenes and terpenoids (Figure 5c). The fluorescence of the MOF nanosheets was quenched upon exposure to chiral terpenes/terpenoids through supramolecular interactions between the chiral binding sites of the MOF nanosheets and gaseous analytes. The chiral nature and precise arrangement of the binding sites facilitated enantioselective fluorescence quenching (Figure 5d) [141]. Although fluorescent sensors based on organic materials exhibit superior selectivity, the widespread deployment of VOC detectors is fundamentally hindered by their system‐level complexities. The requirement for advanced optical instrumentation, particularly precise hardware necessary to differentiate and measure the excitation and emission light, presents a significant challenge to miniaturization and cost‐effective integration, in contrast to chemiresistive or simple colorimetric platforms. Furthermore, mitigating the photodegradation of fluorescent probes is crucial for ensuring long‐term stability, as high‐energy light is continuously irradiated for excitation.

#### 3.3.3 Plasmonic Sensors

Localized surface plasmon resonance (LSPR) occurs when the frequency of the incident light aligns with the oscillation frequency of the conduction band electrons in gold or silver nanoparticles. This alignment significantly enhances light absorption and scattering at frequencies that excite LSPR, resulting in unique extinction spectra influenced by the characteristics of the nanoparticles and the refractive index of their external environment. Consequently, alterations in the dielectric environment near the nanoparticle surfaces, such as molecular adsorption, manifest in the extinction spectrum. LSPR has garnered significant interest owing to its capability for noninvasive and label‐free detection of VOCs [498, 499, 500]. Organic [239, 240, 259] and inorganic materials [241, 334, 343, 346] have been used to augment the VOC‐sensing capabilities of LSPR‐based sensors. These materials are engineered to regulate the transfer of analytes to the LSPR surface, thereby improving the sensitivity and selectivity of the system. Chen et al. employed molecularly imprinted sol–gel (MISG) sieving layers to differentiate volatile terpenes using LSPR. MISG, an inorganic counterpart of molecularly imprinted polymers (MIPs), is synthesized in the presence of target analytes to adjust the pore size and selectivity. Their objective was to distinguish between typical plant biomarkers, including *cis*‐jasmone, limonene, α‐pinene, and γ‐terpinene, using these terpenes as templates for the formation of TiO_2_‐based MISG layers. Gold nanostructures equipped with MISG layers derived from different terpene templates demonstrated selectivity toward identical terpene vapors of their templates [334]. Surface‐enhanced Raman scattering (SERS) is a spectroscopic technique that relies on LSPR. The inelastic scattering of light by molecules is significantly enhanced when these molecules are adsorbed onto corrugated metal surfaces, such as silver and gold nanoparticles. SERS is particularly advantageous for complex odor recognition because of its ability to produce high‐dimensional, molecule‐specific vibrational fingerprints. These fingerprints serve as ideal multidimensional datasets for pattern recognition driven by ML [501]. Although SERS can generate fingerprints of trace analytes at low concentrations near the surface, gaseous analytes must be trapped near SERS‐active surfaces to produce detectable signals (Figure 6a). However, weak interactions between analytes and metals hinder the capture of VOCs in detectable quantities on surfaces; therefore, SERS necessitates condensation methods near SERS‐active surfaces for VOC sensing [500, 502, 503]. MOFs [270, 271, 292, 375, 424, 426, 440], layered double hydroxides (LDHs) [269, 308, 309], MXenes [310, 311], and other inorganic materials [293, 425] have been employed as concentrators upstream of SERS‐active surfaces to amplify the signals of the target VOCs. Qiao et al. utilized gold superparticles as SERS‐active surfaces and coated them with a MOF (ZIF‐8) layer to detect gaseous aldehydes with high sensitivity (Figure 6b). The well‐defined pores of the MOF layer selectively sieved the analytes and influenced their flow owing to size effects and interactions (Figure 6c,d). Consequently, the MOF layer extended the adsorption time of VOCs on the SERS‐active surfaces and enhanced their signal intensities, enabling the ppb‐level detection of gaseous aldehydes [270]. Although pre‐concentrating porous overlayers enhance sensitivity, they impede analyte transport owing to interactions between the porous walls and analytes. Consequently, there exists an inherent trade‐off between pre‐concentration efficiency and the response/recovery kinetics when utilizing these overlayers [424, 440]. Shi and Fang addressed this issue by employing yolk–shell structures to improve the sensitivity and response/recovery speed of SERS sensors. They developed a MOF (ZIF‐8) layer to encapsulate the gold cores, followed by etching the MOF to create hollow structures. The hollow configuration of the MOF layer facilitated more efficient analyte diffusion than the pristine MOF layers, thereby achieving a faster response. Additionally, the hollow structures permitted analytes to access the gold core interfaces, thereby increasing the effective SERS‐active surface areas and enhancing sensitivity [292]. Hierarchical porous structures, characterized by the presence of pores of varying sizes—namely, micro‐, meso‐, and macropores—hold significant potential for optimizing the sensitivity and response time of VOC sensors [504]. These structures leverage larger pores to facilitate the rapid transport of analytes, while smaller pores enhance surface area and pre‐concentration efficiency, thereby modulating both speed and sensitivity [271, 505, 506, 507]. The gate‐opening effect of flexible MOFs represents a promising strategy for addressing the trade‐offs between speed and sensitivity. Flexible MOFs possess the ability to adapt their conformations in response to variations in gas pressure, and these structural modifications can be harnessed for selective VOC adsorption or VOC sensing [508, 509, 510, 511, 512]. Lee et al. introduced a ligand‐mixing strategy to design defects and enhance the gate‐opening effect of MOFs. These flexible MOFs, with deliberately introduced defects, exhibited exceptional adsorption capacity for the hazardous chemical warfare agent simulant, 2‐chloroethyl ethyl sulfide. The ability of flexible MOFs to more readily open their pores holds promise for facilitating the rapid diffusion of analytes within them [509]. Although these advanced material architectures effectively address the kinetic trade‐offs at the sensing interface, plasmonic platforms remain limited by their inherent system‐level complexity. The necessity for large excitation lasers and high‐resolution spectrometers poses a significant challenge to the integration of SERS and LSPR technologies into compact sensing systems. Additionally, the scalable and cost‐effective fabrication of highly reproducible plasmonic nanostructures remains a critical obstacle to their widespread commercial application.

**FIGURE 6:**
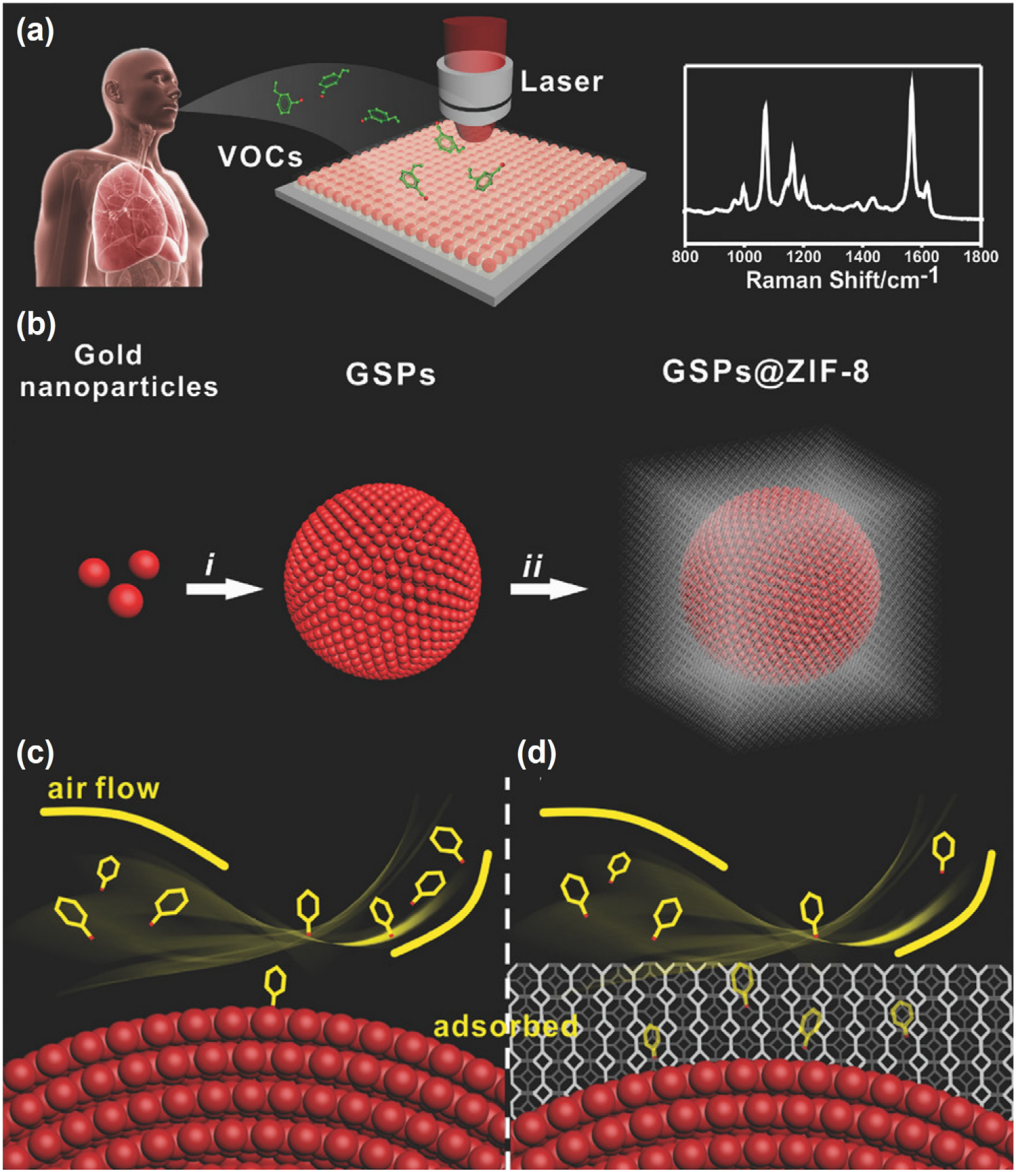
(a) Concept of VOC detection using SERS. (b) Schematic representation of MOF‐coated gold superparticles (GSPs) designed for the sensitive detection of volatile aldehydes. Schematic illustrations of the flow and adsorption of aldehydes on (c) uncoated and (d) MOF‐coated GSPs are shown. Reproduced with permission [270]. Copyright 2017, Wiley.

## 4 Materials and Interfaces for Odor Sensing

In this section, we examine the materials and interfaces of VOC sensors, which play a crucial role in determining their sensing characteristics. Various organic and inorganic materials have been used as transducers and receptors in VOC sensors. Organic materials, such as organic molecules, polymers, MOFs, and COFs, are particularly suitable for receptors because of their precisely regulated and designable chemical structures, which allow tailored selectivity in VOC sensors. These organic‐based receptors have the ability to recognize similar compounds, including structural isomers and enantiomers [142, 143, 252, 278]. In addition, carbon‐based materials such as graphene and CNTs have been introduced. These materials are appealing for broadening the analyte scope and enhancing the performance of VOC sensors owing to their large surface area and the ability to functionalize their termini or immobilize a diverse range of functional materials [197, 513]. Metal and metal oxides have often been employed as catalysts to promote chemical reactions at interfaces and enhance sensing performance by selectively converting analytes into more reactive species [514, 515]. Emerging 2D materials hold promise for sensitive detection with low power consumption, which is attributed to their high carrier mobilities at room temperature and their capacity for surface functionalization [516]. However, their sensing targets have predominantly been confined to small gas species, necessitating further investigation into the detection of larger and more informative volatile organic molecules.

### 4.1 Metals and Metal Oxides

Metals and metal oxides are extensively utilized as sensing materials in chemiresistors because of their thermal and chemical stability, which facilitates heating to enhance surface reactions and achieve a high response to analytes [451, 517, 518, 519]. A diverse array of metal oxide semiconductors, such as ZnO, SnO_2_, NiO, WO_3_, and Co_3_O_4_, has been employed in chemiresistors to convert the surface adsorption and reactions of VOCs into changes in resistance [520]. Furthermore, metals and metal oxides are frequently used as catalysts to enhance the selectivity, sensitivity, and response/recovery speeds by promoting the surface reactions of gas analytes. Noble metals, including palladium, platinum, and gold, have been incorporated into metal oxide chemiresistors to improve their sensing properties for H_2_, NO_2_, CO, and other small gas species [517, 521, 522, 523, 524]. Kim et al. functionalized SnO_2_ nanowires with metal nanoparticles (Pd, Pt, or Au) and integrated them into chemiresistors. The metal catalyst‐modified surface reactions imparted distinct selectivity to the SnO_2_‐based chemiresistors. The Pd‐decorated sensor exhibited the highest response to benzene, whereas the Pt‐decorated sensor was most sensitive to toluene [399]. Recently, metals and metal oxides have been employed as filters to enhance the selectivity and sensitivity of sensors. These catalytic filters selectively remove or deactivate interference while activating the target analytes [514, 522]. Broek et al. implemented an activated alumina filter upstream of a chemiresistor to facilitate the selective detection of exhaled isoprene in human breath. Human breath contains ethanol, acetone, and ammonia, which can interfere with the detection of hydrophobic isoprene because of their high concentrations and interactions with the sensing materials. The activated alumina filter preferentially adsorbs hydrophilic interferences, thereby slowing their transport to the sensing component. Conversely, the hydrophobic isoprene was not adsorbed by the filter and was rapidly conveyed to the sensor. Consequently, the selective adsorption filter enabled nonspecific Pt‐doped SnO_2_ chemiresistors to selectively detect isoprene at low ppb concentrations [72]. The Lee group employed catalytic overlayers on chemiresistors to tailor the reactivity of the target VOCs and interference with the sensors. For example, they used catalytic overlayers (Cr_2_O_3_ or Pd‐V_2_O_5_‐TiO_2_) as filters for the selective detection of ethylene, which is a representative plant hormone (Figure 7a). The selective detection of ethylene is challenging because of its lower reactivity than that of interferents such as ethanol, formaldehyde, and amines, which are prevalent in real‐world sensing environments. Catalytic overlayers can reduce the sensor response to interfering gases by converting them into non‐reactive or less reactive species, such as CO_2_ and H_2_O. In contrast, the catalytic overlayers maintained or even enhanced the sensor response to ethylene by reforming stable ethylene into reactive acetaldehyde (Figure 7b,c) [376, 377]. They also used catalytic overlayers composed of Au, Rh‐TiO_2,_ and CeO_2_ to detect volatile aromatic hydrocarbons in the air. Aromatic hydrocarbons, particularly BTEX, are chemically stable and difficult to detect despite their toxicity. The catalytic overlayers suppressed the sensor response to interfering gases, such as ethanol and formaldehyde, while maintaining a response to the aromatic hydrocarbons. Thus, these catalytic overlayers enable the chemiresistors to selectively detect aromatic hydrocarbons [400, 401, 402]. For instance, Rh‐TiO_2_ catalysts enabled the underlying SnO_2_ chemiresistor to detect benzene with a higher sensor response than toluene and *p*‐xylene (Figure 7d). Although the Rh‐TiO_2_ catalysts transformed benzene, toluene, and *p*‐xylene into different chemical species, their conversion rates and products depended on the chemical stabilities of the reactants. The catalytic overlayer reformulated benzene into a reactive species, such as acetaldehyde, to enhance the sensor response. In contrast, the catalysts converted toluene or *p*‐xylene into more reactive species, which were ultimately oxidized to non‐reactive or less reactive species (CO_2_ and H_2_O) at temperatures above 350°C (Figure 7e). This is because toluene and *p*‐xylene are inherently more reactive than benzene because of the presence of methyl groups adjacent to the aromatic ring. Therefore, the Rh‐TiO_2_ catalysts facilitated the selective detection of benzene by reforming benzene into more reactive species, while toluene and *p*‐xylene were completely oxidized into inactive species [401]

**FIGURE 7:**
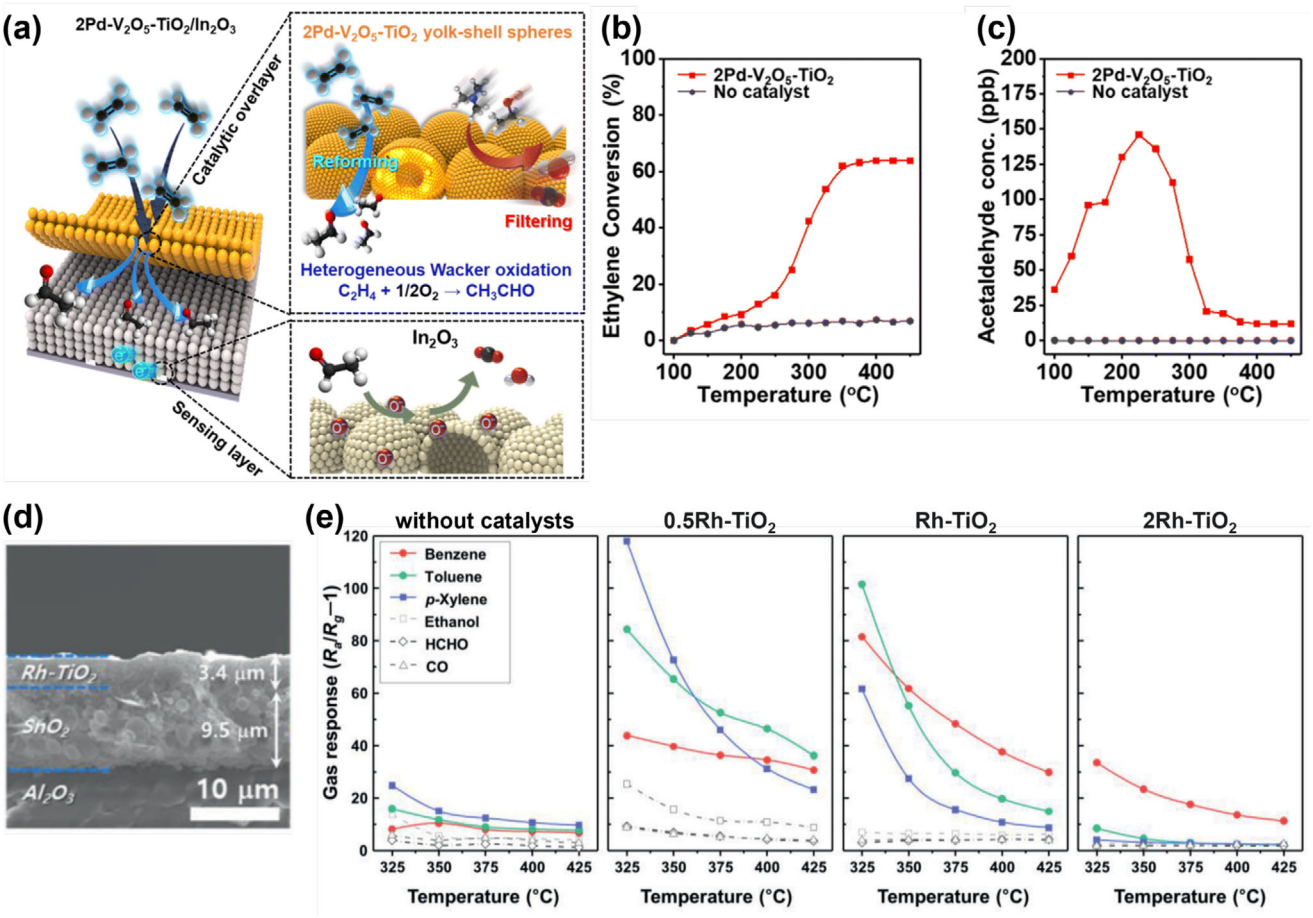
(a) Schematic representation of the mechanism facilitating selective ethylene sensing via a catalytic overlayer. (b) Catalytic efficacy of the Pd–V_2_O_5_–TiO_2_ catalyst for ethylene conversion at different temperatures. (c) Ethylene and acetaldehyde concentrations in the outlet gas with and without the catalyst. Reproduced with permission [376]. Copyright 2022 Royal Society of Chemistry. (d) A cross‐sectional scanning electron microscopy (SEM) image of the sensing film integrated with the catalytic overlayer. (e) VOC sensing characteristics of SnO_2_ chemiresistors with and without the Rh‐TiO_2_ catalytic overlayer, where the catalyst compositions were adjusted. Reproduced under terms of the CC‐BY license [401]. Copyright 2021, Moon, Y. K.; Jeong, S.‐Y.; Jo, Y.‐M.; Jo, Y. K.; Kang, Y. C.; Lee, J.‐H., published by Wiley.

### 4.2 Organic Molecules

Organic molecules are frequently employed as receptors to enhance the sensing performance of specific gaseous targets. These organic receptors can be synthesized to tailor their chemical structures and specific interactions, which are crucial for achieving high sensitivity and selectivity in distinguishing between similar VOCs. Cavitands, such as cyclodextrins [98, 111, 344, 435, 443], calixarenes [213, 228, 368, 373, 409, 434], pillararenes [288, 327, 391, 394], and others [374, 410, 525], have been extensively utilized for selective molecular recognition because their small and well‐defined sensors modified with cavitands can accurately identify structural isomers and stereoisomers using host–guest chemistry. Clément et al. employed deep cavitand moieties as receptors for chemiresistors to detect benzene with high sensitivity and selectivity. Their chemiresistor demonstrated a greater response to benzene than to toluene or *o*‐xylene, detecting benzene at 2.5 ppb at room temperature, with a calculated limit of detection (LOD) of 600 ppt (0.6 ppb), indicating significantly high detectability for benzene [410]. Porphyrins have been used as receptors owing to their designable π‐conjugated ring structures and metal centers. The π‐conjugated systems of porphyrins can interact with the hydrocarbon skeletons of VOCs, and their metal centers can strongly capture coordinating VOCs. Consequently, porphyrin‐based materials have been applied in QCM [142, 214, 247, 369, 431, 444], chemiresistive [133, 154, 183], and FET [353, 416, 417] sensors for the detection of VOCs. Natale et al. developed a QCM sensor array based on metalloporphyrins to determine the storage time of fish by analyzing the patterns of VOC release. Metalloporphyrins provide various responsive properties by altering their metal centers (Ru, Sn, Co, or Mn), making them suitable materials for cross‐reactive sensor arrays [214]. Metalloporphyrins possess axial binding sites and exhibit color changes owing to the formation of coordination bonds between their metal centers and VOCs. Consequently, metalloporphyrins have been extensively utilized in colorimetric sensors for the detection and discrimination of VOCs based on their color change patterns [231, 289, 302, 526, 527]. SAMs are ordered and oriented monolayers of organic molecules that serve as powerful tools for functionalizing sensing components owing to their facile modification and exceptional stability [448, 528]. SAMs have been employed to tailor the sensing properties of electrical VOC sensors. Material combinations for SAM formation, such as gold‐thiol [66, 173, 245, 261, 407, 430, 529], silicon oxide‐silane [206, 207, 208, 209, 530], and metal oxide‐phosphonic acid [313, 314, 531] systems have been investigated. Nakamura et al. modified ZnO nanowire‐based chemiresistors with octadecyl phosphonic acid (ODPA) to enhance the recovery time of sensors during gaseous nonanal sensing. Nonanal, a typical lung cancer biomarker, was oxidized to its corresponding carboxylic acid, which was strongly adsorbed onto the ZnO surface of the sensor. The stability of the carboxylates produced on the unmodified ZnO surface led to sensor irreversibility, which is widely known as catalyst poisoning, resulting in a prolonged recovery time (Figure 8a). In contrast, the ODPA‐modified ZnO chemiresistor detected nonanal in a reversible manner and with higher sensitivity than the unmodified chemiresistor (Figure 8b). The destabilized carboxylates were desorbed from the surface at lower temperatures, enabling the reversible sensing and rapid sensor recovery of the ODPA‐modified ZnO sensor (Figure 8c) [314]. SAMs can also be applied to other detection techniques as they provide defined and dense monolayer structures to tailor surface functions [158, 239, 255, 290, 532, 533, 534, 535]. Li et al. functionalized gold nanoparticles or nanorods with cysteine and used them as plasmonic aggregative colorants. Gold nanomaterials with cysteine exhibit color changes upon exposure to (*E*)‐2‐hexenal, a late blight biomarker. The colorimetric sensor array based on gold nanomaterials and conventional organic dyes classified ten individual plant volatiles and enabled the diagnosis of tomato late blight as early as two days post‐inoculation [255].

**FIGURE 8:**
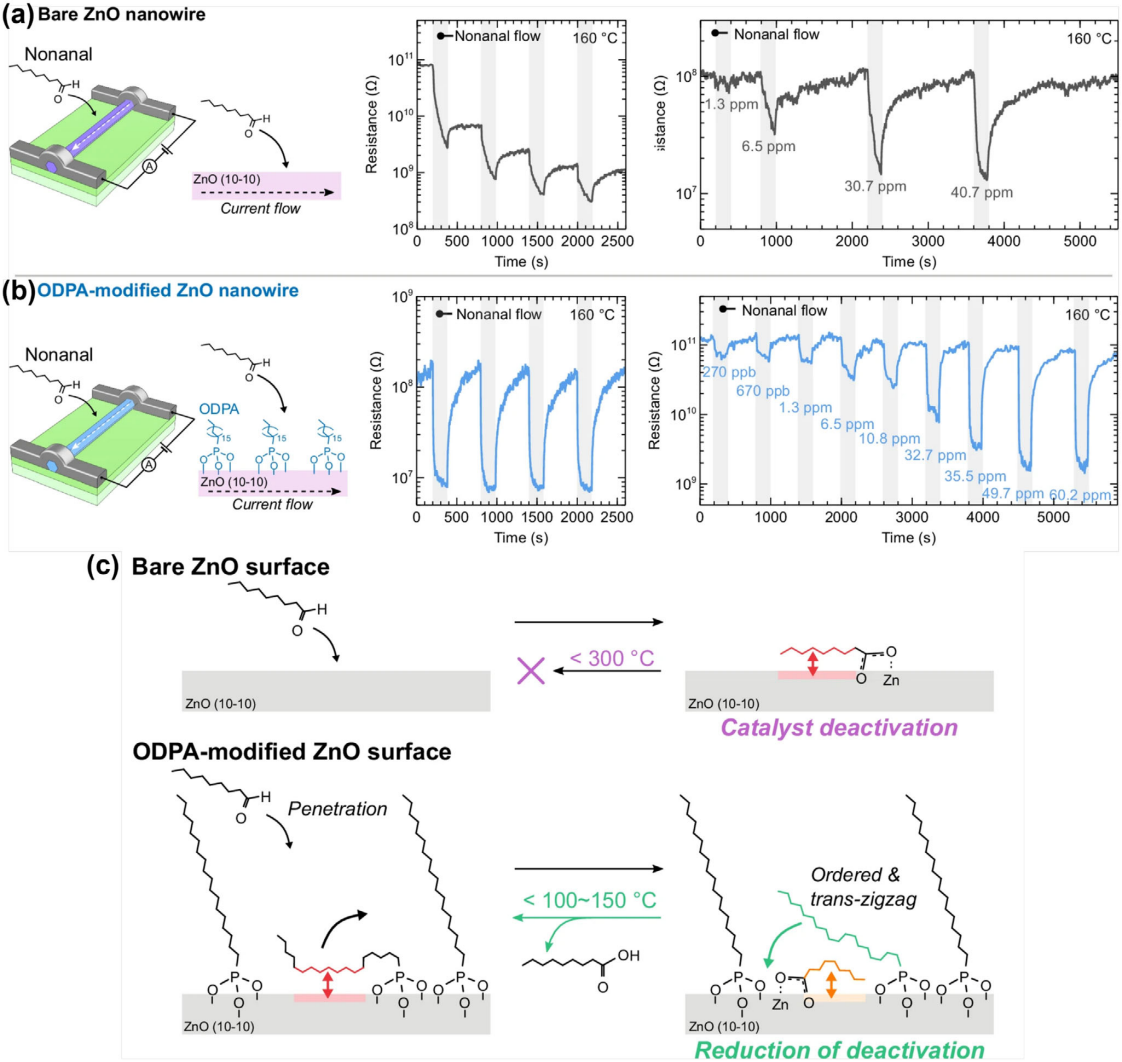
The schematic illustrates the sensing surfaces of (a) unmodified and (b) ODPA‐modified ZnO nanowire sensors. Additionally, the sensing profiles of these sensors upon exposure to nonanal are presented. (c) A mechanism is proposed for the suppression of catalyst poisoning through the ODPA modification during aldehyde sensing on the ZnO surface. Reproduced with permission [314]. Copyright 2024, Springer Nature.

### 4.3 Polymers

Polymers have been employed as sensitizers for gravimetric sensors because of their ease of coating and ability to modify the chemical or physical properties of the sensors [164, 216, 217, 218, 250, 298, 354, 418, 536]. Julian et al. developed a polymer‐coated QCM sensor array as an economical and portable system for VOC detection. Commercially available polymers, such as polyacrylonitrile, poly(vinylidene fluoride), poly(vinyl pyrrolidone), and poly(vinyl acetate), have been applied to QCM sensors to produce distinct sensor response patterns for VOCs. The QCM sensor array successfully differentiated benzene, toluene, xylene, and aliphatic primary alcohols with varying alkyl chain lengths, demonstrating straightforward and effective functionalization achieved through polymers [216]. Conductive polymers or polymer matrices integrated with conductive fillers (polymer nanocomposites) can function as chemiresistive or FET sensors [176, 177, 179, 262, 408, 411, 433, 537, 538]. Conductive polymers alter their resistance by interacting with gaseous analytes [128, 178, 539, 540]. Li et al. used conductive polythiophene polymers to construct a chemiresistive sensor array. Modifications to the side and end groups of the polymers influenced their sensing properties, leading to cross‐reactivity in the sensor array for VOC discrimination [178]. Polymer nanocomposites adjust their resistance based on the distance between the internal conductive fillers, which is affected by the swelling of polymer matrices [337, 538, 541]. Luo et al. developed a chemiresistor utilizing pentiptycene polymer/single‐walled carbon nanotube (SWCNT) composites with a high surface area. The pentiptycene moieties and SWCNTs were interconnected through noncovalent π–π interactions, resulting in the formation of planar cavities. These planar cavities within the polymer/SWCNT composites confer selectivity toward benzene, toluene, or *o*‐xylene, which possess planar aromatic rings that conform to the shape of the cavities [412]. Wang et al. integrated calixarene‐substituted polythiophene with SWCNTs to fabricate chemiresistors and QCM sensors capable of discriminating between xylene isomers (*o*‐, *m*‐, and *p*‐xylene). The calixarene moieties within polythiophene capture VOCs, and their adsorption capacities are influenced by the steric hindrance of VOCs. Consequently, sensors based on polythiophene/SWCNT composites demonstrated a heightened response to *p*‐xylene, which has a smaller molecular volume than other isomers [434]. MIPs are promising materials for VOC sensors owing to their customizable selectivity based on the lock‐and‐key mechanism. MIPs are synthesized by polymerizing functional monomers and crosslinkers in the presence of template molecules. The structural information of the templates, including their functional groups or skeletal shapes, is imprinted onto the MIPs as functional monomers, and crosslinkers are polymerized around the templates. Subsequently, the templates were removed, leaving precisely designed cavities for molecular recognition. These molecularly imprinted cavities are formed to selectively capture templates because of the precise alignment of the interactive sites that reflect the chemical structures of the template molecules [542, 543]. VOC sensors functionalized with MIPs have demonstrated selectivity toward target gaseous analytes including aromatic hydrocarbons [436], terpene/terpenoids [276, 347, 445], alcohols [544], aldehydes [299, 300, 301, 316], and carboxylic acids [341]. Chen et al. synthesized hexanal‐selective MIPs using methacrylic acid, ethylene glycol dimethacrylate, and hexanal template. The MIP‐equipped QCM sensor selectively detected aliphatic aldehydes among other VOCs, including alcohols, carboxylic acids, ketones, and amines. Furthermore, MIPs exhibited a greater frequency shift toward hexanal than octanal or nonanal, as molecules with longer alkyl chains did not fit the MIP pores due to steric hindrance [300]. Wen et al. employed an MIP‐coated QCM sensor array to discriminate terpene vapors and diagnose insect infestation in citrus fruits. They functionalized QCM sensors with six MIPs synthesized using D‐limonene, α‐pinene, myrcene, linalool, β‐ocimene, and decanal templates, which are characteristic molecules associated with insect infestation. QCM sensors demonstrated molecular recognition by MIPs and detected insect infestations [276].

### 4.4 COFs

COFs are porous materials constructed using organic linkers. They have gained prominence as a compelling category of organic materials because of their substantial specific surface area, high stability, adjustable porosity, and customizable functionalities [545, 546]. The regulated porosity and adsorption sites of COFs have been exploited in QCM, [161], chemiresistive [242, 350, 363, 547], and chemicapacitive [415] sensors. Yuan et al. developed a COF‐based chemicapacitor for the detection of benzene. The COF film, characterized by its controllable thickness and high crystallinity, demonstrated a significant benzene adsorption capacity, attributed to the strong COF–benzene interactions. The framework distortion induced by benzene adsorption increased the dielectric constant of the COF thin film, facilitating selective benzene detection at room temperature, with a detection limit of 340 ppb [415]. COFs have also been employed as fluorescent sensors for VOCs because of their atomically precise structures, which provide notable selectivity, and the inherent sensitivity of fluorescence to minor changes in the chemical environment [278, 360, 362, 395]. Wu et al. synthesized chiral fluorescent COFs to differentiate chiral VOCs using fluorescence quenching. They incorporated enantiopure 1,1′‐bi‐2‐naphthol (BINOL) moieties into chiral COFs to form nanofiber membranes that functioned as fluorescent sensors. The fluorescence of these chiral COF‐based membranes was quenched upon exposure to chiral VOCs, including α‐pinene, limonene, fenchone, carvone, and terpinen‐4‐ol. Supramolecular interactions between the chiral BINOL moieties and chiral VOCs resulted in fluorescence quenching of the COFs, with quenching ratios exhibiting enantioselectivity [278].

### 4.5 MOFs

MOFs are porous materials formed through coordination bonds between metal nodes and organic ligands in their structure. These structures offer substantial specific surface areas and can be finely tuned in terms of topology, porosity, and reactivity to external stimuli. Consequently, MOFs are frequently utilized in gravimetric sensors to enhance their sensitivity and selectivity for VOCs [219, 221, 252, 277, 421, 446, 548, 549, 550, 551, 552]. The Heinke group synthesized homochiral MOFs on QCM sensors for the enantioselective detection of chiral VOCs, including enantiomers of limonene, 2‐octanol, 1‐phenylethanol, 1‐phenylethylamine, and methyl lactate. They adjusted the pore sizes of the homochiral MOFs and demonstrated the significant influence of pore size on the enantioselectivity of the sensors. The pore sizes of MOFs determine the accessibility of VOCs to their chiral centers, which is crucial for chirality recognition. Thus, the pore characteristics of MOFs govern the dominant interactions between MOFs and VOCs, significantly affecting their selectivity for molecular adsorption and QCM response (Figure 9a) [253, 447, 553]. Electrically conductive MOFs have potential applications in chemiresistors, although research on a limited number of gas species has been conducted [185, 281, 379, 554, 555]. Campbell et al. developed a cross‐reactive chemiresistor array based on conductive MOFs. They employed three conductive MOFs with different metal centers and ligands to modify their chemiresistive responses. The MOF‐based chemiresistor array classifies VOCs into molecular categories: alkanes, alcohols, ketones/ethers, aromatic hydrocarbons, and amines [185]. MOFs are also advantageous for colorimetric and fluorescence‐sensing applications. They uniformly immobilized dyes or fluorophores and concentrated VOCs through their precise size‐controlled pores, resulting in enhanced sensitivity [235, 267, 357, 556, 557]. Shen et al. incorporated fluorescent probes into MOFs (ZIF‐8). The dispersion and confinement of fluorophores within MOFs leads to a higher fluorescence quantum efficiency by suppressing aggregation‐caused quenching (ACQ) owing to intermolecular interactions between the fluorophores. Fluorophores confined in MOFs can sensitively detect diethyl chlorophosphite, a neurotoxic analog, through wavelength shifts in the enhanced fluorescence. MOFs also served as protective layers to improve the selectivity, photostability, and thermal stability of fluorophores (Figure 9b) [557]. MOFs can function as filtration layers and reaction promoters in VOC sensors to enhance selectivity and sensitivity [171, 270, 280, 319, 349, 413, 558]. Yao et al. combined a MOF (ZIF‐CoZn) with ZnO‐based chemiresistors for the selective and sensitive detection of acetone in exhaled breath. ZIF‐CoZn facilitated the passage of acetone while preventing interference, such as H_2_O, from reaching the underlying ZnO chemiresistive surface. Furthermore, the ZIF‐CoZn‐modified ZnO chemiresistor exhibited a higher sensor response to acetone than the unmodified chemiresistor. This sensitization is attributed to the catalytic activity of ZIF‐CoZn, which generates active oxygen species on the underlying ZnO surface to enhance its reactivity (Figure 9c) [319].

**FIGURE 9:**
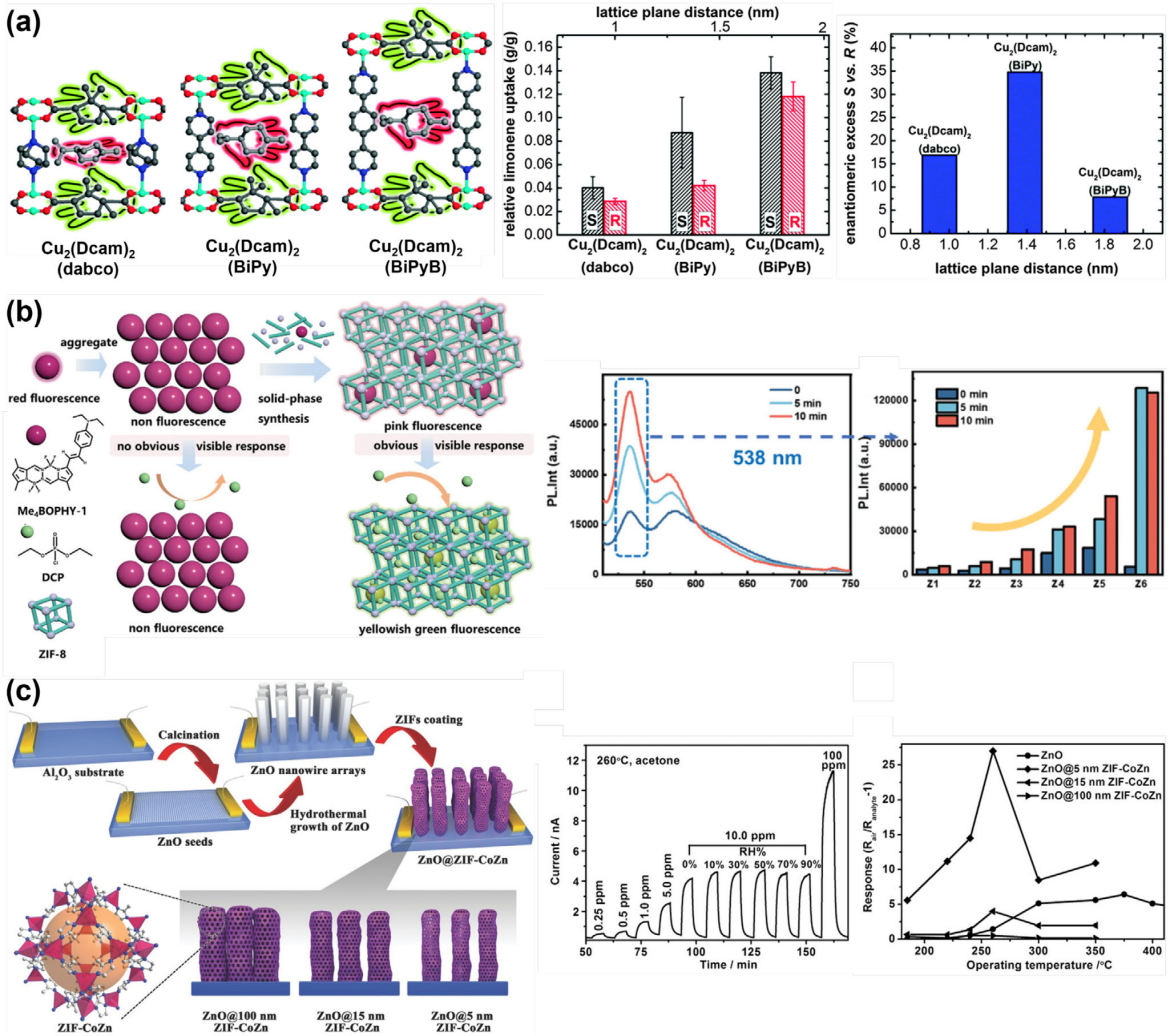
(a) Schematic representations of (*S*)‐limonene adsorbed at the pore centers of homochiral MOFs of the type Cu_2_(Dcam)_2_(L), where L represents diazabicyclo[2.2.2]octane (dabco), 4,4′‐bipyridyl (BiPy), and 1,4‐bis(4‐pyridyl)benzene (BiPyB). Despite the identical chiral moieties (depicted as hands) within the frameworks, variations in pore size influenced enantioselectivity. Reproduced with permission [447]. Copyright 2015, Royal Society of Chemistry. (b) Schematic representations of fluorophore‐embedded MOFs and their high‐performance fluorescent sensing capabilities for diethylchlorophosphite. Reproduced with permission [557]. Copyright 2024, Wiley. (c) Schematic representation of the synthesis of ZnO@ZIF‐CoZn chemiresistors. The MOF films improved the humidity resistance and acetone sensitivity of the ZnO‐based chemiresistor owing to their hydrophobic properties and catalytic activity, respectively. Reproduced with permission [319]. Copyright 2016, Wiley.

### 4.6 2D Materials

Two‐dimensional (2D) materials have emerged as promising nanomaterials for gas‐sensing applications primarily because of their ability to detect VOCs with exceptional sensitivity at low temperatures. This capability is attributed to their large specific surface area and high carrier mobility. Furthermore, 2D materials serve as loading matrices to immobilize functional receptors, thereby enhancing sensor properties [559, 560, 561, 562]. Consequently, 2D materials, such as graphene [118, 186, 197, 320], MXenes [193, 244, 294, 335, 364, 432, 563], 2D MOFs [141, 185, 238, 256], 2D COFs [278, 279, 312, 355], and TMD, [78, 254, 367], have been employed in VOC sensors. Graphene has been extensively utilized for VOC sensing because of its high sensitivity to adsorbed molecules and facile functionalization capabilities. Composed entirely of surface atoms, graphene offers high specific surface area and significant conductance changes in response to chemical environments [564]. Graphene can be functionalized through surface atom engineering [118, 187] or by incorporating a variety of materials, including organic molecules [188, 197], polymers [263], peptides [140, 565], DNA [210, 306, 566], proteins [273], salts [567], and metal‐oxide nanostructures [427, 428]. Chen et al. employed a reduced graphene oxide (RGO) film as a scaffold for gold nanoparticles in a SERS sensor. The RGO film selectively adsorbed and concentrated volatile biomarkers in the breath, enabling sensitive SERS detection without interference from H_2_O or CO_2_. Raman bands were utilized for principal component analysis (PCA) to distinguish between patients with early or advanced gastric cancer and healthy controls [260]. MXenes, which are early transition metal carbides, carbonitrides, and nitrides, exhibit high conductivity and have abundant active sites. MXenes are generally represented by the formula M_n+1_X_n_T_x_, where M denotes early transition metals, X denotes carbon or nitride, and T denotes surface terminal groups. The predominant method for synthesizing MXenes involves etching the corresponding MAX phases, typically formulated as M_n+1_AX_n_, where A represents elements from groups 13 or 14. The etching process selectively removed element A from the parent MAX phases, generating surface terminal groups [568, 569, 570]. MXene‐based composites with organic molecules [244, 311, 571], metals/metal oxides [294, 295, 335, 429, 563], and other materials [87, 365, 366] have been developed for VOC sensing. Yao et al. introduced MnO_2_ catalysts on the surface and between the layers of Ti_3_C_2_T_x_‐MXene for hexanal sensing. The fabricated MnO_2_/Ti_3_C_2_T_x_‐based chemiresistor demonstrated selective detection of hexanal with high sensitivity at 100°C, which was attributed to the formation of p–p heterojunctions and their high catalytic activity [294]. 2D MOFs and COFs have been employed to enhance the sensing properties because their interactions with VOCs can be tailored through their reactive sites and pore characteristics. Chen et al. incorporated chemosensitive dyes into 2D Zn_2_(bim)_4_ nanosheets to amplify the sensitivity of the dyes to VOCs. The dispersion of the dyes on the 2D Zn_2_(bim)_4_ nanosheets mitigated the aggregation‐induced inactivation of the dyes. Furthermore, the large surface area and abundant unsaturated metal sites of the 2D MOF nanosheets improved their capture capacity for VOCs [256]. Evans et al. utilized 2D COF films on QCM sensors by spray‐coating boronate‐ester‐linked COF colloidal inks. The 2D COF films exhibited high sensitivity to volatile organic amines because of their defined porosity and Lewis‐acidic boron atoms. QCM sensors functionalized with 2D COF films can detect trimethylamine at concentrations as low as 10 ppb [355]. TMDs are another class of 2D materials with the formula MX_2_, where M refers to transition metals and X represents chalcogens. TMDs are attractive materials for VOC sensing applications with low power consumption because they have large surface‐to‐volume ratios and tunable semiconducting carrier transport properties that depend on the number of atomically thin layers [572, 573]. Theoretical studies have indicated the potential of TMDs to detect larger VOCs, including aliphatic aldehydes, which are a significant class of volatile biomarkers [574, 575, 576]. However, the scope of gas‐phase sensing via TMDs has been predominantly limited to the more sensitive or selective detection of small gas species at lower temperatures [191, 192, 577]. Gaggiotti et al. employed a QCM sensor array modified with 2D TMD nanosheets to detect large molecules in air. Bulk powders of MoS_2_, WS_2_, MoSe_2_, and WSe_2_ were exfoliated into 2D nanosheets in water using ellagic acid, tannic acid, catechin, and sodium cholate. The liquid‐phase exfoliation process simultaneously functionalized the TMD nanosheets, resulting in the preparation of 16 distinct nanosheets derived from the combination of four TMDs and four organic molecules. The TMD‐modified QCM sensor array exhibited distinct response patterns toward alcohols, terpenes, esters, and aldehydes [254].

### 4.7 Other Materials

CNTs have attracted significant attention because their conduction channels are composed of surface atoms that are sensitive to minor alterations in the chemical environments. Furthermore, CNTs can immobilize a variety of functional materials through covalent and noncovalent bonding mechanisms [513, 578]. CNTs and composites of CNTs with other materials have been extensively employed in the development of chemiresistors and FETs [181, 184, 330, 331, 442, 579, 580]. Wang and Swager advanced this field by functionalizing CNTs with receptors via covalent bonds to create chemiresistive sensor arrays. They employed diverse organic receptors, such as hexafluoroisopropanol, crown ether, calixarene, and long‐chain dodecyl groups, to engineer the distinct selectivity of the sensor components for VOCs. Chemiresistors, based on covalently modified CNTs, were integrated into a sensor array capable of discriminating VOCs and classified into five categories: aliphatic hydrocarbons, aromatic hydrocarbons, chlorinated hydrocarbons, alcohols, ethers, and ketones [181]. Kybert et al. fabricated FETs utilizing CNTs functionalized with DNA oligomers to discern subtle molecular differences between the structural isomers and enantiomers of terpenes. DNA oligomers were attached to the CNTs through noncovalent π–π interactions. These DNA‐functionalized FETs successfully discriminated between the enantiomers of limonene and the three pinene isomers. Although these terpenes consist solely of hydrogen and carbon atoms, the multiple interactions between DNA oligomers and these VOCs facilitated distinctly different sensor responses [442]. ILs, comprising organic cations and organic or inorganic anions present promising sensing interfaces owing to their customizable functionalities [225, 304, 581, 582]. The facile synthesis of various ILs by tailoring ion pairs complements the cross‐reactive sensor arrays for VOC discrimination [104, 226, 304, 583, 584]. Park et al. developed a chemiresistor array based on CNTs and ILs. The combination of cations and anions in ILs was tailored to enhance the chemiresistive response to VOCs, thereby providing cross‐reactivity and capability for VOC discrimination within the sensor array [304]. The Chu group designed ILs to detect specific gaseous analytes based on typical organic chemistry reactions. For example, Schiff base reactions, strain‐promoted [3 + 2] azide‐alkyne cycloadditions, and Diels‐Alder reactions have been employed to detect gaseous carbonyl compounds (aldehydes and ketones), azides, and alkenes, respectively. QCM sensors functionalized with ILs can selectively detect target analytes through interactions or reactions [283, 284, 285, 323, 585].

## 5 Challenges for Smelltronics

VOCs containing multiple carbon atoms serve as highly informative markers and are advantageous for various applications including health diagnostics, food inspection, and environmental monitoring [62, 120, 586]. Significant challenges must be addressed to improve stability, speed, selectivity, and sensitivity (4s) of sensors to allow accurate VOC sensing in real‐world and to extract valuable information [587]. Although VOCs with several carbon atoms and heteroatoms (e.g., amines, alcohols, aldehydes, carboxylic acids, and esters) are indicative compounds, they are challenging targets for reliable sensing [9, 289]. Because these VOCs tend to strongly interact with the sensing materials, preventing quick response/recovery and reversible operation, and impairing the long‐term stability of the sensors. Additionally, these VOCs are often found in mixtures with similar compounds owing to their biological and industrial production pathways. Water vapor is a common and problematic interference in real‐world sensing environments, particularly in biological sample analysis. It is abundant in the atmosphere and biofluids, and frequently exhibits a strong affinity for sensing surfaces [261, 315, 385, 588, 589]. Therefore, the distinguishability or selectivity of sensors must be improved for the precise identification and quantification of VOCs. VOCs with larger molecular weights are present at low concentrations in the gas phase because of their limited production and strong intermolecular interactions. Furthermore, despite their significance, volatile aliphatic/aromatic hydrocarbons and terpenes present detection challenges because of their lack of distinct functional groups for interactions or reactions with sensing materials [72, 233, 377, 400]. Consequently, the detection limits of VOCs must be enhanced to address these deficiencies in terms of their concentration, interaction, and reactivity. Another critical consideration, other than 4s, is compatibility with low‐power and miniaturized platforms. Given the potential of smelltronics for continuous and noninvasive monitoring of VOCs emitted by humans, animals, plants, and other living organisms, it is advantageous to integrate sensors into mobile or wearable devices [590, 591, 592]. Although sensor arrays, often referred to as e‐noses, are promising platforms owing to their redundancy and discrimination capabilities, the integration of heteromaterials poses challenges to low‐power operation, miniaturization, and diversity of sensing interfaces. Pretreatment components, such as filters, micro‐gas chromatography (µGC), and pre‐concentrators, have been incorporated into sensors to enhance their performance; however, these additions are disadvantageous because of their effects on power consumption and miniaturization (Figure 10a,b). In this section, we discuss the key challenges in smelltronics and the methodologies for enhancing the performance of VOC sensors.

**FIGURE 10:**
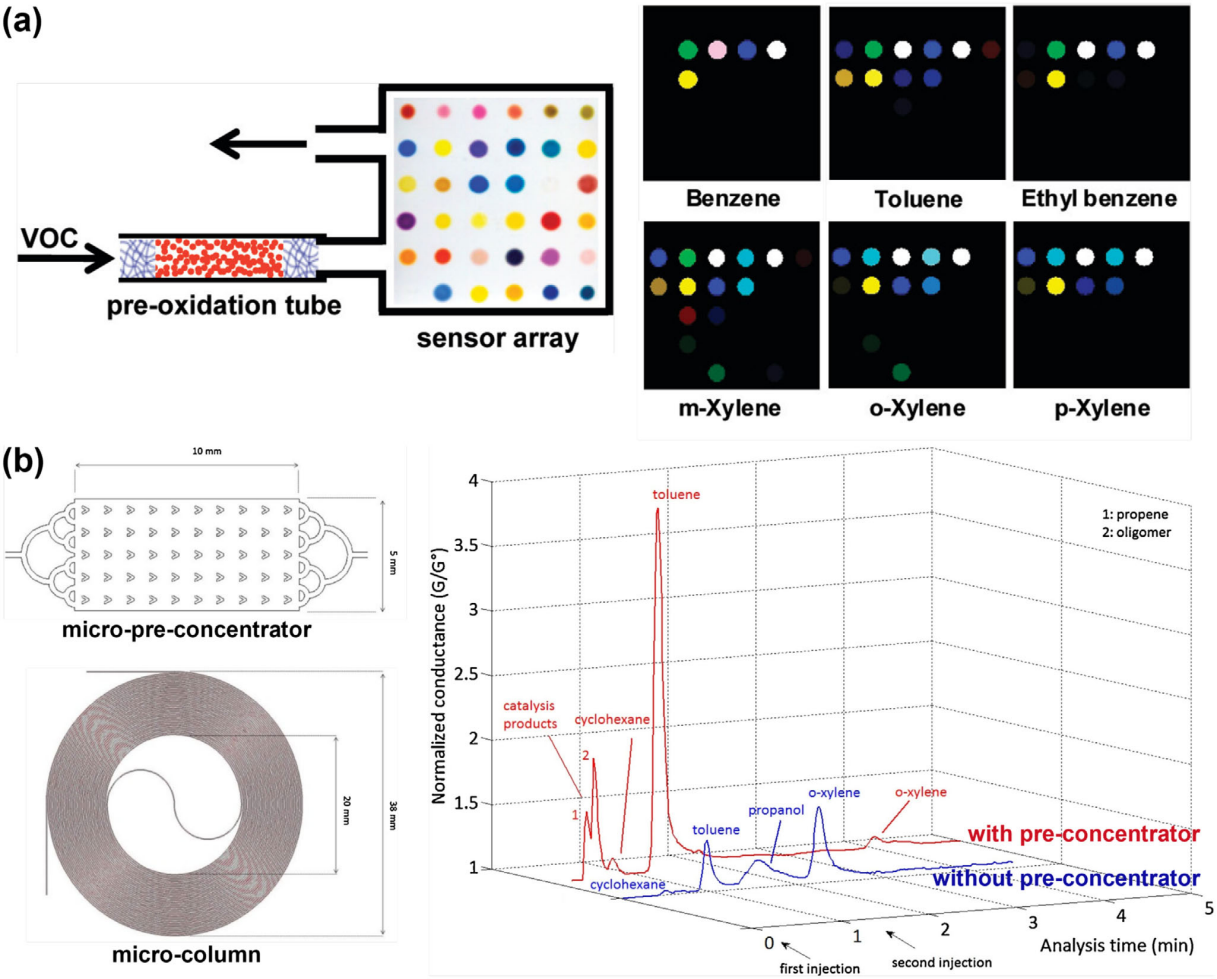
(a) Schematic representation of the pre‐oxidation tube positioned upstream of the colorimetric sensor array, designed to enhance the detectability and distinguishability of the sensor array. Reproduced with permission [233]. Copyright 2011, American Chemical Society. (b) Diagrams of the miniaturized pre‐concentrator and separation column for the analysis of VOC mixtures at low concentrations. The time‐course chemiresistive responses to pre‐concentrated and separated VOCs are also depicted. Reproduced with permission [382]. Copyright 2018, Elsevier.

### 5.1 Long‐Term Stability

The long‐term stability of VOC sensors is crucial for reliable and continuous monitoring of VOCs, as variations in their sensing characteristics impede the collection of time‐series data necessary for AI‐driven chemical big‐data analysis [593]. Achieving stability in sensing materials presents an inherent challenge, as these materials must consistently undergo and reverse changes through interactions with chemical species to generate specific signals. This requirement creates a fundamental trade‐off between the stability and responsiveness of the sensing material. In addition, during the sensing operation, the characteristics of the VOC sensors are altered by two primary factors: incomplete recovery and sensor drift. Incomplete recovery arises from irreversible binding or interactions of analytes with the sensing surface. Sensor drift, on the other hand, is caused by various external influences, such as atmospheric gas species, humidity, thermal effects, and bias stress [169, 384, 594, 595, 596]. Ensuring the stability and reproducibility of sensors necessitates meticulous fabrication and long‐term experiments, which often deter researchers from focusing on these issues [378, 597, 598, 599, 600, 601, 602, 603]. Various sensing materials have been investigated to enhance the sensitivity and selectivity of VOC detection. Although large surface areas, molecular confinement, and multipoint interactions are advantageous for discriminating chemical species, these beneficial properties can degrade the reversibility of the sensor [140, 462, 493, 554]. Therefore, comprehensive optimization of the sensing materials, devices, and systems is necessary to ensure reliable VOC sensing over an extended period. Interfacial engineering, grounded in transduction mechanisms, is particularly essential in the stable operation of nanoscale sensors because the heterointerface properties of nanomaterials dominate the overall device performance [593, 604]. In terms of a robust sensing system, drift compensation is enthusiastically investigated due to the advancements in information science, although it is still inadequate for addressing unforeseen degradation mechanisms or environmental variations [169, 170, 605, 606, 607]. An alternative approach for achieving long‐term stable VOC sensors involves refreshing sensor characteristics by applying external stimuli, such as heat, voltage, and light [608, 609, 610, 611, 612, 613, 614]. This methodology is beneficial for maintaining the response properties of devices and analysis algorithms. Liang et al. utilized the photocatalytic activity of TiO_2_ to promote the oxidative degradation of several organic compounds. They employed porous thin films of TiO_2_ as optical sensors for monitoring dodecane, isopropanol, pentane, and water. The low volatility of these VOCs leads to irreversible adsorption, impairing baseline reproducibility. However, UV irradiation during the recovery process accelerated the decomposition and desorption of adsorbed species, resulting in a fully recovered baseline [610]. The long‐term stability and reversibility of sensors are particularly critical when analyzing informative VOCs with several carbon atoms, as strong interactions between volatile species and surfaces impede their desorption and the recovery of the sensing characteristics.

### 5.2 Discrimination of Complex Mixtures

For practical applications of VOC sensing, it is imperative that the sensors identify and quantify specific species within mixtures. This task is particularly challenging because of the complexity of real‐world environments and the inherent cross‐reactivity of the sensors. For example, human breath contains numerous VOCs that can indicate health status. These volatile biomarkers are specific to particular diseases, underscoring the importance of accurately detecting each chemical species [35, 37, 63]. GC is a widely utilized and effective technique for analyzing VOC mixtures, as it separates the components of complex mixtures based on the varying affinities between the adsorbing materials and analytes [615, 616]. µGC is a miniaturized separation system that facilitates the analysis of VOC mixtures using portable sensors [168, 318, 329, 382, 404]. Zampolli et al. successfully quantified volatile aromatic hydrocarbons, such as benzene, toluene, ethylbenzene, and *m*‐xylene, from their mixtures by employing a miniaturized pre‐concentrator and separation column. They achieved this by etching silicon wafers to create suitable shapes for a pre‐concentration or separation unit, which was then coated with adsorbing materials. A quinoxaline‐bridged cavity was used as the pre‐concentrator, and a commercially available stationary phase for GC was used as the separation column. Aromatic hydrocarbon mixtures were pre‐concentrated using cavitands, separated through a miniaturized chromatographic column, and subsequently detected using a metal‐oxide‐based chemiresistor array. Consequently, the chemiresistor was able to identify and quantify each aromatic hydrocarbon target in their mixtures at sub‐ppb concentrations [404]. Gregis et al. detected four volatile biomarkers for lung cancer using a single SnO_2_‐based chemiresistor equipped with a miniaturized pre‐concentrator and separation column (Figure 10b) [382]. Zhang et al. developed an origami hierarchical sensor array capable of separating VOCs on paper using a chromatography‐like approach. The hierarchical structure formed by multiple layers of paper controlled the VOC transport toward the sensing layers. The gaseous analytes were delivered to each sensor layer by passing through the paper layers, generating time‐space‐resolved responses that resulted in highly discriminative patterns of resistance change in the sensor layers. These sensor response patterns enabled the discrimination of the structural isomers and chiral enantiomers of VOCs [189]. Although separation techniques that mimic GC are highly effective, a more biomimetic approach to odor sensing involves the direct quantitative monitoring of individual components of unseparated VOC mixtures. In most fundamental studies, researchers typically assess the performance of sensors against a single target gas in a clean background to elucidate the intrinsic interactions and sensing mechanisms. However, in practical environments, sensor responses are significantly influenced by the co‐existence of diverse VOCs and interferents, as individual sensors rarely exhibit absolute selectivity. Consequently, addressing this inherent cross‐sensitivity without prior physical separation is crucial for rapid and low‐power device operation in the future. To achieve this, researchers have increasingly integrated cross‐reactive sensor arrays with advanced ML algorithms. By training regression models with multidimensional signal matrices, overlapping responses can be mathematically deconvoluted to accurately quantify multiple target gases simultaneously [167, 197]. In this context, multivariable sensors, such as FETs, are particularly advantageous. A single multivariable sensor can generate multiple independent signals, thereby significantly reducing the number of sensors required in an array while enhancing the overall system robustness and reliability [209, 475]. Furthermore, dynamic modulation techniques, such as operating a single sensor under varying temperatures [317, 617] or light irradiation [618], generate temporally resolved high‐dimensional data that reflect the unique adsorption/desorption kinetics of different co‐existing gases. This dynamic approach provides a powerful method for the quantitative monitoring of component gases in complex mixtures without the need for bulky physical separation units.

### 5.3 Humidity Interference

Humidity is a significant challenge in VOC detection. This is attributable to the abundance of water molecules in real‐world environments and their strong interactions with sensing materials. For example, human breath exhibits a relative humidity (RH) of 80%, corresponding to over 25 000 ppm of water vapor at 1 atm and 25°C. Given that most VOCs of interest in ambient air or human breath are present at ppm or ppb levels, humidity inhibits the adsorption or interaction of target VOCs with the sensing materials [261, 619]. Consequently, humidity obscures the sensor response to the target VOCs, thereby diminishing the discriminatory capabilities of sensors [180, 385]. The utilization of sensing materials on hydrophobic scaffolds, such as hydrophobic silica nanoparticles [118, 264, 300], silica plates [231, 371], membranes [234, 286, 494], and other substrates [256, 265], enhances the humidity resistance of the sensors. Chen et al. synthesized hydrophobic amino‐functionalized graphene oxide (AGO) nanocomposites by integrating AGO with hydrophobic silica nanoparticles (HSNPs). These nanocomposites were used in QCM sensors to detect VOCs emitted from fish fillets. The amino groups on the AGO facilitate the selective detection of volatile aliphatic aldehydes, such as hexanal, octanal, and nonanal, which serve as indicators of fish freshness. The incorporation of hydrophobic silica nanoparticles enhanced the humidity resistance of the QCM sensors, enabling reliable VOC detection at 80% relative humidity (RH) (Figure 11a) [118]. The polarity of the sensor scaffolds also influences the sensor selectivity. Soga et al. developed simple colorimetric sensor arrays by incorporating amine‐responsive dyes into polymer nanoparticles with varying polarities. Each sensor array component encapsulated identical dyes in mixtures of hydrophobic and hydrophilic polymers at different ratios. Upon exposure to aliphatic primary amines, the sensor arrays exhibited distinct color patterns corresponding to polarity matching between the volatile amines and polymer scaffolds [356]. To achieve distinct molecular selectivity while maintaining robust resistance to humidity, the creation of tailored molecular pockets has proven highly effective. For instance, Chen et al. synthesized hydrophobic MIPs by integrating them with hydrophobic silica nanoparticles. When applied to QCM sensors, these precisely tailored molecular cavities demonstrated the remarkable capability to discriminate hexanal from closely related homologues (octanal and nonanal), while simultaneously suppressing background responses from ambient humidity and other polar interferents [300]. Coating sensing components with hydrophobic materials is another effective strategy for enhancing humidity resistance, although it may affect the sensitivity or selectivity of the sensor. Hydrophobic polymers [194, 215, 267, 348], inorganic materials [620, 621, 622], SAMs [571], MOFs [319], and COFs [266] have been used as water filtering layers in sensing devices. Wang et al. dispersed dyes on MOFs (UiO‐66(Zr)) and coated the resulting dye/MOF composites with hydrophobic polydimethylsiloxane (PDMS). MOFs increase dye sensitivity by providing a high surface area and pre‐concentrating VOCs. The PDMS coating prevented water molecules from entering the dye/MOF composites while capturing VOCs. A colorimetric sensor array was fabricated using PDMS‐coated dye/MOF composites and mounted on the surface of a wheat leaf to monitor the emitted VOCs. The sensor array successfully discriminated plant diseases under highly humid conditions based on the colorimetric changes induced by VOCs (Figure 11b) [267]. An alternative approach involves the use of an external dehumidifier to selectively remove water molecules, thereby improving humidity resistance without altering the sensing material. Liu et al. developed an external dehumidifier using inorganic nanowires to detect VOCs under humid conditions. They prepared ZnO/TiO_2_/CaCl_2_ core‐shell heterostructured nanowires and positioned them upstream of chemiresistors. When exposed to VOCs in humid environments, the ZnO/TiO_2_/CaCl_2_ nanowires adsorbed water molecules while allowing gaseous analytes such as ethanol, acetone, nonane, 2‐nonanone, and nonanal to pass through. Water‐selective adsorption by the nanostructured dehumidifier enhanced the sensitivity of the sensor to gaseous analytes in the presence of moisture (Figure 11c) [315]. The surface‐refreshing methodology is advantageous for humidity‐tolerant VOC sensors [351, 623]. Wu et al. utilized CeO_2_ nanoparticles in chemiresistors composed of Ti_3_C_2_T_x_ MXene/carbon nanofiber composites. Environmental water molecules alter the surface conditions, such as the quantity of oxygen vacancies and hydroxyl groups, thereby impairing the sensor's response. However, CeO_2_ nanoparticles, which exhibit coexisting Ce^3+^ and Ce^4+^ states, can induce redox reactions with water molecules that are adsorbed on their surfaces. This mechanism effectively refreshes the surface conditions even in 80% RH environments, resulting in a humidity‐independent chemiresistive response to dimethylamine [351]. Another promising material design strategy involves the utilization of hierarchical porous structures to strategically control molecular diffusion [624]. Verma et al. developed a Sn‐SnO_2_ nanocomposite by integrating Sn nanoparticles with SnO_2_ nanospheres. The mesoporous and microporous morphologies of these nanospheres facilitated the efficient diffusion and confinement of nonpolar BTEX molecules within the nanostructures. This architectural design significantly enhanced the sensing response to BTEX, while effectively minimizing cross‐sensitivity to highly polar interferences and ambient moisture [625].

**FIGURE 11:**
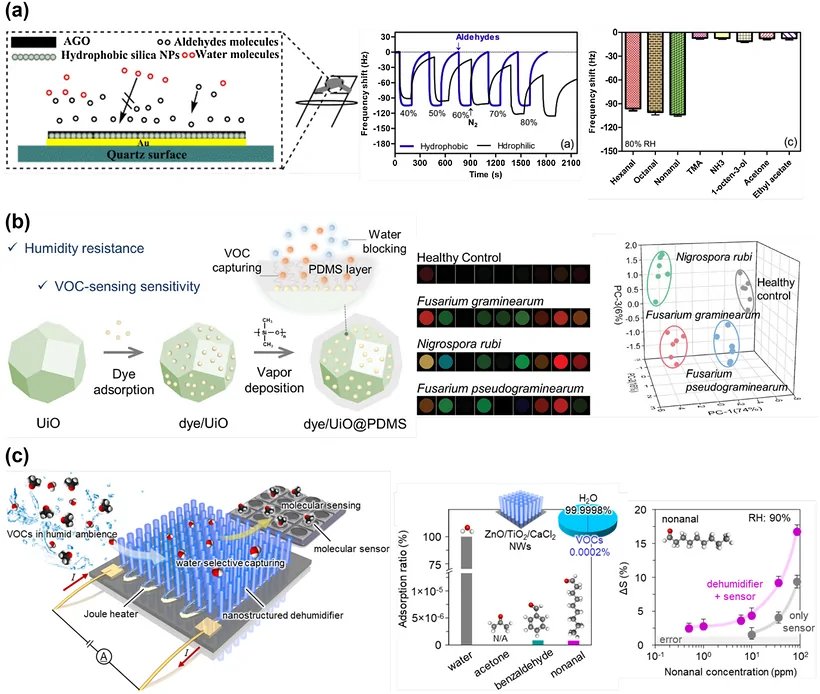
(a) A QCM sensor incorporating hydrophobic AGO nanocomposites selectively detects volatile aldehydes and exhibits high humidity resistance. Reproduced with permission [118]. Copyright 2020, Elsevier. (b) A schematic representation of the dye/UiO@PDMS core‐shell gas‐sensing composites, illustrating the removal of water by the PDMS layer for VOC detection. The results of the analysis of VOCs emissions from plants are also presented. Reproduced under terms of the CC‐BY license [267]. Copyright 2024, Wang, X.; Qi, H.; Shao, Y.; Zhao, M.; Chen, H.; Chen, Y.; Ying, Y.; Wang, Y., published by Wiley. (c) A schematic of the external dehumidifier, which comprises ZnO/TiO_2_/CaCl_2_ core‐shell heterostructured nanowires, is provided. The water‐selective adsorption properties of the inorganic dehumidifier and the enhancement of the sensor sensitivity to nonanal at 90% RH are also shown. Reproduced with permission [315]. Copyright 2022, American Chemical Society.

### 5.4 Low‐Reactivity of VOCs

VOCs, particularly volatile hydrocarbons, present significant challenges in detection because of their limited interactions or reactivity with sensing materials. As these VOCs are inherently nonpolar, they primarily rely on weak and nonspecific van der Waals forces for adsorption onto sensing materials and require higher energy to undergo chemical reactions than polar VOCs. Consequently, the transformation of less‐reactive targets into reactive species has emerged as a promising strategy for enhancing the surface adsorption and reactions of VOCs. Therefore, catalysts can be employed in VOC sensors to activate specific molecules or functional groups with high selectivity in complex environments [296]. The application of catalysts to enhance the sensing properties of gas sensors has been investigated, particularly for ethylene [359, 376, 380], isoprene [73, 383], and benzene [150, 399, 405, 406], because these volatile hydrocarbons are significant biomarkers or pollutants, despite the challenges associated with their detection. Fong et al. utilized Wacker oxidation for sensitive and selective chemiresistive detection of gaseous ethylene. They created a catalytic mixture by dissolving bis(benzonitrile)palladium(II) dichloride and tetrabutylammonium nitrite in low‐volatility benzyl alcohol. This catalytic mixture was applied to a chemiresistor to facilitate the aerobic oxidation of ethylene to acetaldehyde via the Wacker oxidation reaction. This catalytic oxidation enhances the sensitivity and selectivity of the chemiresistor by converting ethylene into acetaldehyde, a more reactive species. By adjusting the composition of the catalytic mixtures and chemiresistors, highly sensitive detection of ethylene was achieved with a LOD of 15 ppb [380]. Activation methods are also advantageous for colorimetric sensor arrays. Suslick et al. developed a pre‐oxidation method to activate VOCs and enhance the response of colorimetric sensor arrays. The gaseous analytes were converted into more reactive species when they passed through a tube filled with chromic acid. The pre‐oxidation process amplified the interactions between the oxidized VOCs and the sensing materials, as the dyes changed color based on the local polarity, Brønsted acidity/basicity, Lewis acid‐base interactions, and redox reactions. Thus, the pre‐oxidation method facilitated chemical interactions or reactions between the analytes and dyes, improving the detectability and discrimination power of the colorimetric sensor arrays (Figure 10a) [233, 286, 626].

### 5.5 Detection of Trace‐Level VOCs

The low concentration of VOCs in real‐world environments poses a significant challenge for practical sensor applications as it diminishes their detectability and discrimination capabilities [385]. Although enhancing sensitivity can improve sensor detectability, highly sensitive sensors are also susceptible to interference. Consequently, pre‐concentration techniques are advantageous for augmenting detectability without altering the sensors, and they can also enhance selectivity by designing interactions to capture analytes [627]. External pre‐concentrators have been utilized upstream of sensing components, and their adsorption/desorption behaviors are often thermally controlled. These pre‐concentrators capture and concentrate gases at lower temperatures, subsequently releasing and transporting the concentrated gases to the sensing component by applying heat [248, 382, 403, 404, 558, 628, 629, 630, 631]. Trzciński et al. synthesized a cavitand receptor with quinoxaline walls linked by ethylenedioxy bridges and employed it as a pre‐concentrator. This cavitand receptor pre‐concentrates BTEX by capturing it through host–guest interactions. Subsequently, higher concentrations of BTEX gases were released by applying heat and detected by a downstream photoionization detector (PID), achieving ppb‐level detection of benzene in the air [632]. Lvova et al. utilized CNTs modified with porphyrin units as a pre‐concentrator for the detection of 1‐butanol. The porphyrin‐modified CNTs demonstrated a high affinity for 1‐butanol, as shown by QCM analysis. The combination of a sensitive QCM sensor and a pre‐concentrator employing the same porphyrin‐modified CNTs exhibited a high response, capable of detecting 1‐butanol at ppb levels [248]. Porous materials with high affinities for target analytes and specific surface areas are suitable as pre‐concentrators, with MOFs being representative materials for concentrating VOCs [558, 633, 634]. Leidinger et al. employed MOFs to pre‐concentrate benzene for sensitive detection using commercially available sensors. The pre‐concentrator was fabricated by depositing MOFs (MIL‐53) onto an alumina heater substrate and integrating it with a metal‐oxide semiconductor sensor. The MOF‐based pre‐concentrator effectively trapped and released benzene by controlling the temperature, enabling a commercially available gas sensor to detect benzene at a low concentration of 10 ppb [558].

### 5.6 Integration With Information Technology

Although this review primarily focuses on material innovations aimed at enhancing physical sensing capabilities, the integration of these interfaces with advanced computational tools is indispensable for deciphering the complex VOC mixtures encountered in practical environments. The increasing demand for rapid and reliable on‐site monitoring, encompassing clinical health diagnostics, food quality assessment, and environmental tracking, emphasizes the necessity of combining physical sensor architectures with robust data interpretation frameworks. The translation of raw sensor responses into distinct outcomes typically follows a four‐stage pipeline: signal preprocessing, feature extraction, data labeling, and implementation of ML models. Initially, the raw time‐series outputs undergo conditioning through noise reduction, baseline stabilization, and normalization. Subsequently, the critical steady‐state, transient, and frequency‐domain characteristics are extracted from the response curves to construct the input matrices required for algorithmic training. ML methodologies are broadly categorized into supervised and unsupervised techniques. Supervised learning relies on extensively labeled datasets in which input matrices are explicitly linked to known target variables. These models are trained to classify specific VOC signatures and quantitatively predict analyte concentrations. Supervised learning algorithms widely adopted in VOC sensing include linear discriminant analysis (LDA), partial least squares regression (PLSR), support vector machine (SVM), random forest (RF), k‐nearest neighbors (k‐NN), and multilayer perceptron (MLP). Conversely, unsupervised learning approaches are designed to autonomously discover hidden structures and intrinsic patterns within unlabeled datasets. Crucially, dimensionality reduction is performed to manage the highly multidimensional data obtained by cross‐reactive sensors. PCA is a standard linear technique used to maximize data variance and visualize broad cluster distributions. For nonlinear dimensionality reduction, t‐SNE has proven to be highly effective in preserving local data geometries, thereby enabling a clear distinction between structurally analogous VOC mixtures. Other common unsupervised techniques include k‐means clustering, hierarchical cluster analysis (HCA), and neural network models. Comprehensive bibliometric analyses indicate that ANN, SVM, and PCA remain the dominant computational techniques integrated with modern gas sensors [635, 636, 637, 638]. Although ML algorithms demonstrate exceptional efficacy in classifying multidimensional sensing data, their “black‐box” nature often obscures the underlying physical chemistry that governs sensor‐analyte interactions. To bridge this gap, the emerging application of explainable AI (XAI) is highly anticipated. XAI not only elucidates algorithmic decision‐making but also provides deeper fundamental insights into the interfacial recognition mechanisms between sensing materials and target VOCs, paving the way for more rational sensor design [639].

## 6 Conclusion and Perspective

### 6.1 Conclusion

The emerging field of smelltronics bridges conventional small‐molecule‐based gas sensing and digital olfaction by focusing on volatile molecules with multiple carbon atoms and heteroatoms (e.g., amines, alcohols, aldehydes, carboxylic acids, and esters) that carry rich odor/smell information. In this review, we first clarified how informative VOCs emitted from humans, foods, organisms, and anthropogenic activities represent multidimensional chemical information that is highly relevant to health diagnosis, food inspection, environmental monitoring, safety and security, and personal authentication. We then summarized representative device platforms for odor sensing, including electrical, gravimetric, and optical sensors, emphasizing their characteristic transduction mechanisms, typical material configurations, and classes of VOCs they are designed to detect. Furthermore, we compared key classes of sensing materials and interfaces—from robust inorganic oxides to highly tunable organic materials, MOFs/COFs, and low‐dimensional materials—and discussed interfacial engineering strategies to manage low concentrations, low volatility, and subtle structural differences among VOCs under realistic conditions. Despite these advances, smelltronics still faces fundamental challenges. Many informative VOCs with several carbon atoms and heteroatoms appear only at low gas‐phase concentrations owing to limited production and strong intermolecular interactions. Hydrocarbons and terpenes lack distinct functional groups that enable selective interactions. In addition, VOC sensing inherently involves trade‐offs between robustness and molecular recognition capability: chemically and thermally robust inorganic semiconductors such as metal‐oxide materials generally provide high stability but offer limited molecular recognition, whereas organic and bio‐derived sensing materials exhibit rich and tunable molecular recognition yet often suffer from insufficient robustness under practical operating conditions [455, 640, 641, 642]. Collectively, these insights and remaining challenges demonstrate that smelltronics is evolving from proof‐of‐concept VOC sensors toward an integrated technology platform for extracting, encoding, and exploiting odor information in real‐world environments.

To overcome these intrinsic limitations of VOC sensing and move smelltronics toward reliable and application‐ready technologies, coordinated advances in sensing materials, devices, and systems are essential. In terms of sensing materials, inorganic materials such as metal oxides are known for their chemical and thermal stability. Recently, it has become increasingly clear that van der Waals interactions between the carbon backbone of VOCs and metal oxide surfaces play a much more significant role in molecular sensing at oxide surfaces than previously recognized [314]. Recent studies have demonstrated that van der Waals interactions between VOCs and metal oxide surfaces can be exploited to distinguish chemical homologues [339, 340]. Although such interactions between volatile analytes and sensing surfaces are subtle, they effectively reflect the properties of the analytes, making them a promising basis for reversible yet distinguishable sensor operations. These emerging insights suggest that by rationally exploiting these subtle but informative interactions, chemically and thermally robust metal oxides could realize much more precise molecular recognition than previously anticipated. Supramolecular chemistry is an effective method for detecting molecular structures via weak interactions. The meticulously engineered structures of receptors (hosts) and their interactions with gaseous analytes (guests) facilitate the recognition of carbon skeletons and reversibility, relying on shape suitability and the summation of weak interactions [391, 410, 643, 644]. Recently, adaptive organic materials capable of altering their structures in response to weak interactions with gaseous species, thereby achieving both reversibility and distinctive selectivity, have been developed [395, 645]. Molecularly imprinted inorganic materials offer an additional strategy for tailoring sensor functionality through precise surface structure design. Molecularly imprinted inorganic materials are synthesized by forming inorganic structures in the presence of organic molecules. Subsequently, the removal of these organic templates results in the creation of nanocavities on the surface that mirror the shape of the templates. These nanocavities are capable of selectively capturing analytes that correspond to their specific shape and size, thereby producing molecular‐selective inorganic materials [334, 646, 647, 648]. In parallel, enhancing the stability of organic materials remains an important direction, because these materials, despite their highly tunable functionalities and sensing performance, generally exhibit lower robustness than inorganic materials. Common degradation factors for organic materials include oxygen, water, chemicals, heat, and light [649]. Bottom‐up grown inorganic–organic hybrid materials, in which robust inorganic frameworks encapsulate or support organic sensing components, provide a promising route to suppress degradation of organic sensing layers while preserving their rich molecular recognition capabilities [650, 651, 652, 653, 654, 655]. Consequently, recent research in organic electronics has focused on engineering organic materials to achieve intrinsic robustness without barrier films, including the modulation of energy levels, side chains, and crosslinking structures. These strategies can prevent interfering species from penetrating or reacting with the organic materials and thereby suppress their degradation [649].

The intrinsic trade‐offs of VOC sensing cannot be overcome by material design alone and instead require strategies that encompass both device‐ and system‐level architectures. A sensor device integrates multiple materials, such as sensing layers, electrodes, substrates, and encapsulation, each serving a distinct role in realizing a specific sensing function. In nanoscale devices, the overall sensing performance is often dominated by the properties of the heterointerfaces formed between these components. Therefore, heterointerface engineering is a key strategy for enhancing the performance of nanomaterial‐based sensors. For example, employing conductive metal oxides, such as indium tin oxide (ITO) and antimony‐doped tin oxide (ATO), as contact electrodes for nanoscale metal oxide chemiresistive sensors significantly improves the long‐term stability of sensing properties under high‐temperature operation in ambient air [593, 656]. This is because conventional metal contact electrodes used in chemiresistive sensors, such as Ti, Cr, and Al, are readily oxidized and become highly resistive under sensor operating conditions, whereas oxide electrodes remain chemically and morphologically stable even in oxidizing atmospheres. Similarly, in peptide‐coated QCM sensors, on‐chip growth of peptide nanowires directly on the resonator surface improves interfacial adhesion, enabling low‐dissipation resonance and, consequently, highly sensitive molecular detection [370]. The importance of such heterointerfaces becomes even more direct in low‐dimensional materials, particularly 2D materials, where interfacial properties can strongly dictate charge transport, adsorption kinetics, and sensing responses at the device scale. For instance, MXene/TMD nanohybrid sensors based on inkjet‐printed Ti_3_C_2_T_x_/WSe_2_ heterostructures have achieved low‐noise, flexible, room‐temperature detection of oxygen‐containing VOCs with markedly enhanced sensitivity compared with pristine MXene sensors, demonstrating how engineered 2D heterojunction networks can simultaneously improve sensitivity, selectivity, and stability in practical VOC sensing platforms [657].

Beyond static device architectures, recent studies have proposed sensor‐system‐level strategies that dynamically modulate the molecular reactivity and functionality of sensing materials during operation by applying external stimuli. Dynamic modulation of sensor functionality by heat [12, 16, 17, 18, 19, 20, 617], light [618, 658, 659], or electric fields [614, 660, 661] serves as an alternative strategy to overcome the intrinsic limitations of specific materials and device structures because these stimuli can reversibly alter the chemical reactivity, adsorption/desorption kinetics, or conductivity of the sensing layers. One notable example is a heaterless, low‐power, and robust sensor array based on self‐Joule‐heating of metal oxide nanofilm channel sensors, in which dynamic control of the sensor surface temperature is used to modulate the chemical reactivity and adsorption–desorption equilibrium of the sensing channel, enabling discrimination of complex odors even when using a homogeneous SnO_2_ across the sensor array [617]. In addition, visible‐light‐modulated Bi_2_S_3_/SnS_2_ heterostructure sensors have realized bifunctional room‐temperature detection—showing ultrahigh responses to NO_2_ under light irradiation while maintaining high selectivity to H_2_S in the dark [618].

### 6.2 Perspective

In light of the insights and challenges previously discussed, we propose a strategic roadmap to propel the field of smelltronics toward the development of highly reliable and application‐ready technologies within the next 5–10 years. This progression is anticipated to occur in two distinct phases, facilitated by coordinated advancements in materials, devices, and systems.

Phase 1: Near‐to‐Mid‐Term Outlook (Current–5 Years)—Hardware Innovation and Dynamic Sensor Architectures

The immediate objective over the next five years is to address the fundamental trade‐off between chemical robustness and precise molecular recognition through hardware‐level innovations. Advances in supramolecular chemistry and adaptive organic materials will facilitate the accurate recognition of carbon skeletons through host–guest shape complementarity and synergistic summation of weak interactions. Additionally, researchers will increasingly leverage subtle van der Waals interactions in chemically and thermally robust metal oxides to achieve unprecedented precision in molecular recognition. Surface‐structure engineering, such as the development of molecularly imprinted inorganic materials with template‐derived nanocavities, will be further refined for the physical capture of specific analytes. At the device level, heterointerface engineering will become essential for enhancing the baseline stability and sensor responsivity. As the limitations of single‐component materials become evident, research will shift toward hybrid architectures and dynamically operated systems. To mitigate the degradation of organic components owing to oxygen, moisture, and heat, the development of bottom‐up inorganic–organic hybrid frameworks, in which robust inorganic matrices encapsulate delicate organic sensors, is expected to become a mainstream strategy. Furthermore, sensor systems will transition from static to dynamic modulated platforms. By applying external stimuli (e.g., heat, light, or electric fields) during operation, researchers can dynamically alter the chemical reactivity and adsorption/desorption kinetics. Techniques such as self‐Joule‐heating arrays and light‐modulated heterostructures enable simple or compositionally homogeneous sensor arrays to achieve multidimensional odor discrimination.

Phase 2: Long‐Term Outlook (5–10 Years)—Software Integration and Sustainable Deployment

In the long term, smelltronics will evolve from isolated proof‐of‐concept sensors to fully integrated low‐power digital olfaction platforms optimized for real‐world environments. Future device architectures will be fundamentally integrated with emerging computational paradigms, including neuromorphic computing, reservoir computing, and Edge AI. To facilitate continuous real‐time monitoring of human health, food quality, and environmental conditions, these systems will perform on‐chip pattern recognition rather than relying on power‐intensive sequential data transmission to the cloud. This on‐chip processing effectively eliminates the energy consumption and processing speed bottlenecks that are inherent to conventional sensor arrays. Crucially, localized data processing via Edge AI will ensure high data confidentiality and security, enabling the widespread deployment of smelltronics in privacy‐sensitive applications such as clinical diagnostics and personal authentication [35, 662, 663, 664]. As these systems transition toward massive, ubiquitous, and potentially disposable deployments, the environmental footprint of the hardware itself emerges as a paramount concern. Beyond the conventional 4S challenges—*Stability*, *Speed*, *Selectivity*, and *Sensitivity—Sustainability* should also be established as the fifth “S” for next‐generation chemical sensor platforms. Sustainability is particularly relevant as sensing technologies are increasingly integrated into ubiquitous, disposable, and wearable devices and systems. While the aforementioned edge computing architectures address the operational power consumption of sensor systems, comprehensive sustainability is not limited to the power consumption of sensor systems but also includes the upstream and downstream environmental impacts of sensor materials and fabrication processes. For example, nanomaterial synthesis and device fabrication related to smelltronics may involve solvent‐intensive routes, hazardous chemicals, energy‐intensive cleanroom steps, and high water consumption [665]. In addition, the end‐of‐life management of sensor devices raises broader concerns related to electronic waste, resource recovery, and lifecycle‐aware materials design [666, 667]. Therefore, future sensor research should pursue not only higher device performance but also greener synthesis, lower‐impact fabrication, and more sustainable device lifecycles.

Overall, these perspectives illustrate that future progress in smelltronics will rely on tightly coupled advances in molecularly designed interfaces, heterointerface‐engineered devices, and dynamically operated sensor systems to address the intrinsic trade‐offs inherent to VOC sensing technology. By unifying these elements into integrated platforms, smelltronics can move beyond proof‐of‐concept demonstrations and deliver robust, low‐power, and information‐rich odor sensing tailored for real‐world environments. We anticipate that such developments will ultimately establish smelltronics as a core technology for the digitalization, interpretation, and utilization of odor information in healthcare, food systems, environmental management, and security applications.

## Conflicts of Interest

The authors declare no conflicts of interest.

## Data Availability

The authors have nothing to report.
